# The impact of endothelial cell death in the brain and its role after stroke: A systematic review

**DOI:** 10.15698/cst2019.11.203

**Published:** 2019-09-25

**Authors:** Marietta Zille, Maulana Ikhsan, Yun Jiang, Josephine Lampe, Jan Wenzel, Markus Schwaninger

**Affiliations:** 1 Institute for Experimental and Clinical Pharmacology and Toxicology, University of Lübeck, Lübeck, Germany.; 2 DZHK (German Research Centre for Cardiovascular Research), partner site Hamburg/Lübeck/Kiel, Lübeck, Germany.

**Keywords:** apoptosis, autophagy, lysosome-dependent cell death, necroptosis, ischemia, vasculature

## Abstract

The supply of oxygen and nutrients to the brain is vital for its function and requires a complex vascular network that, when disturbed, results in profound neurological dysfunction. As part of the pathology in stroke, endothelial cells die. As endothelial cell death affects the surrounding cellular environment and is a potential target for the treatment and prevention of neurological disorders, we have systematically reviewed important aspects of endothelial cell death with a particular focus on stroke. After screening 2876 publications published between January 1, 2010 and August 7, 2019, we identified 154 records to be included. We found that endothelial cell death occurs rapidly as well as later after the onset of stroke conditions. Among the different cell death mechanisms, apoptosis was the most widely investigated (92 records), followed by autophagy (20 records), while other, more recently defined mechanisms received less attention, such as lysosome-dependent cell death (2 records) and necroptosis (2 records). We also discuss the differential vulnerability of brain cells to injury after stroke and the role of endothelial cell death in the no-reflow phenomenon with a special focus on the microvasculature. Further investigation of the different cell death mechanisms using novel tools and biomarkers will greatly enhance our understanding of endothelial cell death. For this task, at least two markers/criteria are desirable to determine cell death subroutines according to the recommendations of the Nomenclature Committee on Cell Death.

## INTRODUCTION

The brain is a metabolically active organ that consumes about 20% of the total energy of the body [1]. At the same time, it is unable to store significant amounts of energy, which makes it uniquely dependent on constant supplies provided by the circulation. For this reason, the brain contains a dense vascular network. Thus, the average distance between microvessels is less than 20 μm in the gray matter, suggesting that each neuron has its own capillary [2]. In clinical medicine, vascular pathology is the most common cause of central nervous system dysfunction presenting in the form of acute stroke or chronic small vessel diseases. As part of these vascular pathologies, endothelial cell (EC) death occurs and affects the surrounding cellular environment, making it a potential target mechanism for the treatment and prevention of neurological disorders. This review provides a systematic overview of important aspects of EC death with a particular focus on stroke.

### Characteristics of brain ECs

Together with neurons and the extracellular matrix, cerebral vessels are part of the neurovascular unit. They are composed of various cell types, including ECs, smooth muscle cells, pericytes, perivascular macrophages, and astrocytes. An intensive crosstalk between cells via soluble factors, including cytokines, contributes to vessel structure, function, and maintenance [3]. The endothelium is the core building block forming the innermost layer of vessels and representing the pioneering cells for new vessels. During development, ECs differentiate from intra-embryonic mesoderm-derived angioblasts and invade the mouse brain by capillary sprouting at around E9.5 [4]. Recently, erythro-myeloid progenitors born in the yolk sac have been reported to differentiate into a second subset of ECs occurring in the mouse brain after E10.5 as well as in other organs [5].

Under the influence of adjacent cells in the neurovascular unit, the endothelium develops the characteristic properties of the blood-brain barrier (BBB). The BBB is a highly selective barrier through which transport is accurately controlled. Accordingly, brain ECs are characterized by a unique expression pattern of genes, including those for various tight junction proteins that restrict paracellular transport [6–9]. More than this, the transcellular transport is limited by low transcytosis rates and specialized transporters that are able to deliver only specific compounds through the BBB and exclude many others. Unfortunately, many of the characteristic *in vivo* features of the BBB are lost when the cells are cultured [10]. Therefore, conclusions based on cell culture studies that we report below should be verified *in vivo*.

### Physiological EC death

During development, the growth of the brain is associated with an expansion of the vascular network by angiogenesis. Intravital imaging has demonstrated a profound remodeling of the vasculature in the developing mouse cortex and zebrafish mesencephalon [11, 12]. Capillaries with low perfusion are shut down, indicating that endothelial proliferation is accompanied by vessel pruning to shape the newly formed vasculature according to tissue needs. Chen and colleagues observed that cell movement drives vessel regression without evident cell death in the brains of zebrafish larvae [11]. However, a more recent analysis demonstrates that some EC apoptosis occurs during the remodeling of the brain vasculature in zebrafish larvae [13]. Therefore, EC death may still contribute to vessel regression and play a crucial role in vascular remodeling during brain development (for review see [14]).

**Figure 1:**
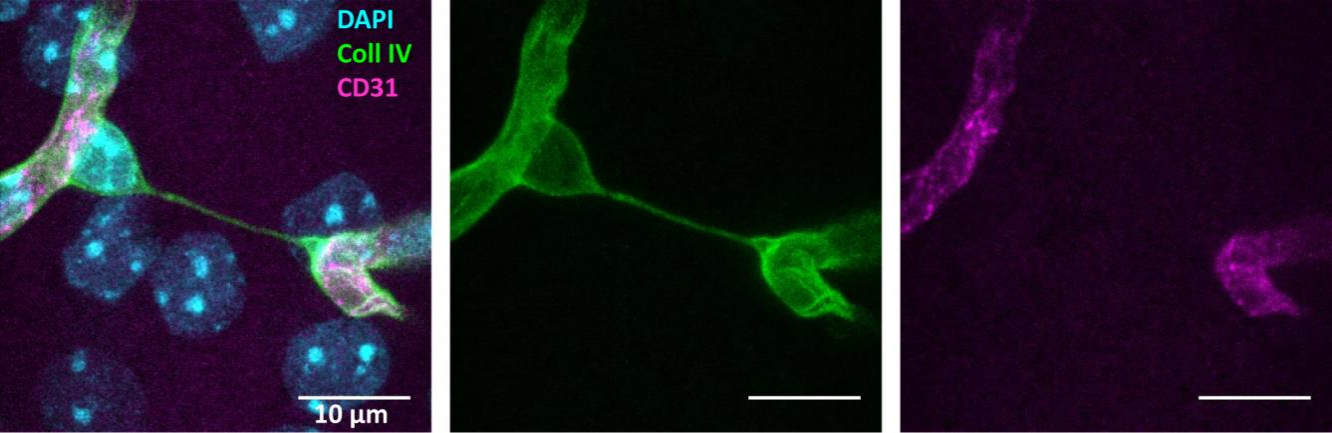
FIGURE 1: Example of a string-vessel in the mouse cortex. The vessels are labeled with the basement membrane marker Collagen IV (Coll IV, green) and CD31 (magenta). String-vessels are positive for collagen IV, but not CD31. Image achieved by confocal microscopy of a 100 μm thick brain slice. Scale bar = 10 μm.

A sensitive measure of vessel loss in brain tissue is the increased formation of so-called “string vessels” **(Figure 1)**. When ECs die, the ensheathing basement membrane is left over and usually collapses to string-like structures. [15, 16]. These acellular capillaries contain collagen IV and laminin α5 as basement membrane components, but lack the pan-endothelium marker CD31 [15]. Laminin α5, a constituent of the endothelial basement membrane, bears witness of the ECs that were once present. Surprisingly, string vessels persist at least for months [15, 16], yielding a cumulative measure of vessel pruning. So far, it is unclear which forms of EC death would give rise to string vessels. Similar structures may even develop in the absence of cell death when, during development, ECs retract from vessels that have a low perfusion [11]. This physiological process may be a partial cause of string vessels that occur throughout the brain under normal conditions [15, 16]. Notably, some string vessels are not covered by pericyte processes and astrocytic endfeet [15]. This feature has led to their interpretation as tunneling nanotubes that are potentially involved in intercellular crosstalk and angiogenesis [17]. Although intriguing, this concept is mostly hypothetical so far.

In adulthood, the cerebrovascular system is quite stable; ECs are quiescent with a long turnover time. This low rate of remodeling further declines with age [12]. There is no evidence for physiological EC death in the brain during early adulthood, but the situation changes in old age. Despite some controversy in the published literature, the majority of studies agree on the existence of brain vessel rarefaction in old age [reviewed in 18]. The density of vessels decreases and string vessels increase with age in humans and animals. A recent study has reported an increase of apoptotic, active caspase-3-positive ECs in the cortex of 20-months old mice [19]. Increased endothelial apoptosis was related to elevated levels of acid sphingomyelinase (ASM) in old mice. ASM triggered apoptosis in the primary culture of ECs. More importantly, EC death and vessel rarefaction were prevented in mice heterozygous for *Smpd1* (the gene encoding ASM) or by pharmacological inhibition of ASM [19]. Thus, ASM appears as a drug target to prevent physiological EC death and its functional consequences.

### The many facets of pathophysiological cell death

While physiological (“programmed”) cell death during development is vital for removing vestigial tissues and for sculpting the body [20], extensive cell death during an injury can be devastating – in particular because brain cells are often irreversibly lost.

For decades, cell death was dichotomized into necrosis and apoptosis based on macroscopic morphological characteristics (morphotypes). Necrosis has been defined by the swelling of organelles, increased volume of cells, and disruption of the plasma membrane, the latter leading to release of intracellular content. In contrast, in apoptosis, cells display signs of cell shrinkage, nuclear fragmentation, chromatin condensation (pyknosis), and chromosomal DNA fragmentation (karyorrhexis) [21, 22]. Subsequently, it became clear that this dichotomy cannot fully explain the complexity of cell death biology. Inhibiting a specific cell death subroutine, such as apoptotic cell death, is not sufficient to prevent cellular demise, including the one that occurs during development, but rather leads to a shift in morphotypes [23, 24].

Until now, a variety of regulated cell death mechanisms have been described. These include anoikis (a variant of intrinsic apoptosis initiated by the loss of integrin-dependent anchorage), autophagy-dependent cell death (cell death dependent on the autophagic machinery), ferroptosis (iron-dependent cell death initiated by oxidative perturbations), lysosome-dependent cell death (cell death with primary lysosomal membrane permeabilization and the involvement of cathepsins), and necroptosis (cell death dependent on mixed lineage kinase domain-like protein (MLKL) and receptor interacting protein kinases (RIPK)), among others (for an extensive review, see [25]). This framework opens the opportunity to further characterize cell death subroutines in neurological diseases and gives the hope that, by identifying the underlying mechanisms, new therapeutic targets will emerge.

### Detection of EC death

To identify dead or dying cells, light and electron microscopy have been traditionally used and still represent valuable tools in cell death research. While light microscopic techniques offer the fast and relatively inexpensive detection of cell death, they do not allow discriminating different cell death subroutines. Ultrastructural information as determined by more laborious electron microscopy, on the other hand, can differentiate between necrosis and apoptosis based on the morphological criteria described above (for a review in stroke see [26]).

However, the detection of other cell death subroutines requires biochemical tools and, hence, many markers and methods have been developed in the past [26]. Their usefulness has to be re-evaluated with the knowledge of all the subroutines because only some markers can discriminate between different forms of cell death. As a general recommendation, the Nomenclature Committee on Cell Death encourages the use of at least two independent markers or criteria to confirm a specific cell death mechanism [27]. We will briefly describe the more specific techniques and their caveats in this section.

Imaging based on the terminal deoxynucleotidyl transferase (TdT)-mediated dUTP nick end labeling (TUNEL) reaction, cleavage of caspase-3, increase in annexin A5, and the expression of the apoptosis regulator (BCL2) protein family have greatly contributed to the investigation of apoptotic cell death. TUNEL detects DNA fragments on a single cell basis both *in vitro* and *in vivo* based on the addition of labeled nucleotide triphosphates to the free 3'-OH termini of double-stranded DNA by TdT [28]. While it is widely used as a marker for apoptosis, there is considerable evidence suggesting that TUNEL cannot reliably distinguish between apoptotic and necrotic cell death [29]: cells displaying morphological features of apoptosis do not always show DNA fragmentation [30] and necrotic cells may be TUNEL-positive [31–33].

Active caspase-3 is a more specific marker for apoptosis than TUNEL staining [25]. However, there is also a caspase-independent form of intrinsic apoptosis [27], and some caspases are involved in pro-survival functions [34] as well as other regulated cell death mechanisms [25]. Besides caspase-3 cleavage, the effect of pharmacological inhibitors of caspases may be indicative of apoptosis. Nonetheless, the inhibition of caspases may not prevent cell death but shift its morphotype, an observation that led to the discovery of necroptosis [23].

Annexin A5 (also called annexin V) specifically binds to phosphatidylserine, which is translocated to the outer leaflet of the plasma membrane during apoptosis [35]. However, phosphatidylserine externalization may not always be associated with cell death [36–38]. In addition, annexin A5 also binds to intracellular phosphatidylserine in case the plasma membrane is disrupted, which occurs during both necrosis and later in apoptosis [22, 39, 40]. Annexin A5 can be combined with markers of disrupted membranes such as ethidium homodimer or propidium iodide (PI) [41] that, by themselves, cannot discriminate between different cell death subroutines. For the *in vivo* application, annexin A5 and/or ethidium homodimer/PI have to be injected intravenously prior to histological evaluation of the animal because tissue preparation otherwise generates a high rate of false-positives due to cutting and treatment with detergents that break open the plasma membrane. Fluorescent and radiolabeled annexin A5 probes are available to investigate cell death noninvasively over time [42–44].

The expression of BCL2 family proteins can hint towards apoptosis. BCL2 associated X (BAX, apoptosis regulator), BCL2 antagonist/killer 1 (BAK1 or BAK), and/or BCL2 family apoptosis regulator (BOK) form pores across the mitochondrial outer membrane and increase its permeability. The permeabilization of the mitochondrial outer membrane is antagonized by the anti-apoptotic members of the BCL2 family, including BCL2 and BCL-X_L_ (also BCL2 like 1 or BCL2L1). The anti-apoptotic BCL2 family members sequester BAX and BAK, but are bound to so-called sensitizers or inactivators, including BCL2 associated agonist of cell death (BAD), BH3 interacting domain death agonist (BID), BCL2-interacting mediator of cell death (BIM, also BCL2 like 11, BCL2L11), and p53-upregulated modulator of apoptosis (PUMA or BBC3) (for review see [25]).

As a specific subtype of intrinsic apoptosis induced by the loss of cell adhesion, anoikis can be identified by similar indicators as apoptosis, i.e. the expression of BCL2 family members that participate in or antagonize mitochondrial outer membrane permeabilization and by the activation of the executioner caspase-3.

Autophagy is characterized by the conjugation of the cytosolic form of microtubule-associated protein 1A/1B-light chain 3-I (LC3-I) to phosphatidylethanolamine. The conjugated protein LC3-II and its recruitment to autophagosomal membranes can be detected by western blotting or immunofluorescence. There are twenty genes regulating autophagy (*Atg*) providing opportunities to test for this form of cell death by knockdown or knockout, although some *Atg* genes have functions unrelated to autophagy [45]. Moreover, the autophagy inducer rapamycin (mechanistic target of rapamycin (mTOR)) and the autophagy inhibitors 3-methyladenine (targeting phosphoinositide 3-kinase, autophagosome formation), bafilomycin A1 (inhibiting endosomal acidification), chloroquine (inhibiting lysosomal function), and mdivi-1 (mitochondrial division inhibitor 1 that targets dynamin-related protein 1 (DRP1)) can be used to assess autophagy. Since cells often induce autophagy in an attempt to adapt to stress, blocking autophagy generally accelerates (rather than delays) cellular demise. Under certain circumstances, autophagy can, however, contribute to cell death or engage in other cell death modalities [25].

In ferroptosis, lipid peroxides are not sufficiently cleared by glutathione peroxidase 4 (GPX4) and accumulate. The dyes C11-BODIPY and Liperfluo provide a rapid measure of overall lipid peroxidation, while liquid chromatography and tandem mass spectrometry analysis are able to detect specific oxidized lipids. With the latter technique, it is possible to assess GPX4 activity using phosphatidylcholine hydroperoxide reduction in cell lysates [46]. In addition, there are a number of genetic and pharmacological inhibitors to test for ferroptosis [46].

For the detection of lysosome-dependent cell death, calpain and cathepsin activity are useful markers. Specific inhibitors of their functions, including calpain inhibitor I (ALLN), endogenous protease inhibitors (cystatins and serpins), pharmacological agents specific for cysteine cathepsins (e.g., E64D and Ca-074-Me) or aspartyl cathepsins (e.g., pepstatin A) may provide further evidence for lysosome-dependent cell death [25].

To identify necroptotic cell death, many studies have shown the activation of the necroptotic machinery including RIPK1, RIPK3, and MLKL. While an increase in total protein level of RIPK1 does not conclusively indicate necroptosis, but rather tumor necrosis factor-induced complex IIb apoptosis [47, 48], RIPK1 autophosphorylation at serine 166 as detected by phospho-specific antibodies is a more appropriate measure of RIPK1 kinase activity and necroptosis [49–51]. In addition, antibodies directed against phospho-RIPK3 and phospho-MLKL [52–54] may enhance the investigation of this cell death mechanism in the future. Pharmacological inhibitors, such as necrostatin-1 (Nec-1, versus its inactive variant Nec-1i) and its more stable form Nec-1s (inhibitors of RIPK1) [23, 55], GSK872 and dabrafenib (inhibitors of RIPK3) or necrosulfonamide (inhibitor of MLKL) allow dissecting necroptosis pathways. However, Nec-1 and Nec-1i, but not Nec-1s, also inhibit indoleamine 2,3-dioxygenase [56], while dabrafenib is a blocker of BRAF in cancer cells [57].

In addition to the above-mentioned markers and techniques that identify specific cell death subroutines (except for single staining of PI and ethidium homodimer), more general cell viability/death measures are commonly employed because they can be used for faster throughput and are not biased towards specific mechanisms.

The 3-(4,5-Dimethylthiazol-2-yl)-2,5-diphenyltetrazolium bromide (MTT) assay is a fast and inexpensive, and hence widely used, colorimetric assay of cell viability. It relies on the conversion of MTT to the water-insoluble formazan by mitochondrial succinate dehydrogenase. The decrease of MTT in cell culture is proportional to the number of dead cells. However, the assay is dependent on mitochondrial respiration and indirectly serves to assess the cellular energy capacity of a cell [58, 59]. Thus, interventions affecting mitochondrial respiration may lead to erroneous results. In addition, the MTT assay does not provide information about a specific cell death mechanism, and caution should be taken when interpreting data from cell lines as proliferation may mask effects. Additional evaluation to confirm cell death is necessary, e.g. by microscopy or another independent cell death measure.

Similar to MTT, the lactate dehydrogenase (LDH) assay is also an unspecific measure of cell death. LDH is a soluble cytosolic enzyme that is released into the culture medium upon cell death due to the damage of the plasma membrane. The magnitude of LDH efflux correlates in a linear fashion with the number of cells damaged [60].

## SYSTEMATIC REVIEW OF EC DEATH IN STROKE

The vasculature plays an essential role for the proper functioning of the brain and is involved in the etiology and pathophysiology of cerebrovascular diseases, and potentially also in other neurological disorders [61]. While there are many excellent reviews on the role of the BBB in health and disease [61, 62], the mechanisms of EC death are less well described. The last review on brain EC death was in 2010 [63], before some of the cell death subroutines have been described. Therefore, this review aims to capture a comprehensive picture of the latest findings on brain EC death mechanisms. To do so, we have performed a systematic screen of the literature on EC death according to the guidelines for Preferred Reporting Items for Systematic Reviews and Meta-Analyses (PRISMA) [64].

### Search Strategy

We searched for publications listed in PUBMED describing cell death of brain ECs. Details on the search strategy are provided in the Supplemental Data. Publications between January 1, 2010 and August 7, 2019 were included. We chose the start date to include studies that were published after the last review on this topic [63]. Data from non-brain ECs, EC progenitors, mixed cultures, and whole brain tissue without EC-specific markers were excluded. Publications containing data on BBB dysfunction and neurovascular remodeling without any data on brain EC death were also excluded. We included *in vitro* and *in vivo* studies but only publications in peer-reviewed journals containing primary data. Review articles, articles without full text accessibility, and non-English articles were excluded.

### Selection of Studies and Data Extraction

**Figure 2:**
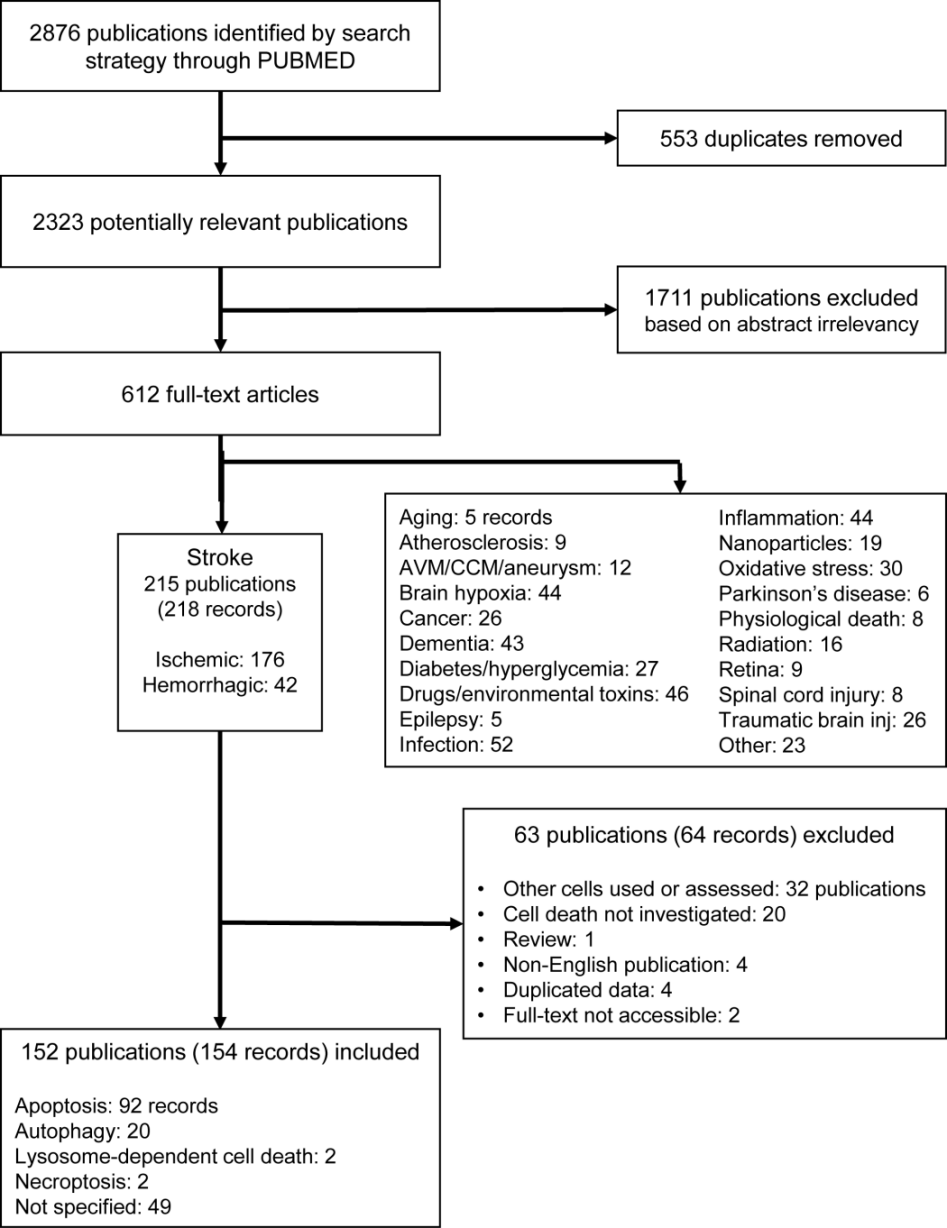
FIGURE 2: PRISMA flow chart of the systematic search on brain EC death. Of note, the number of records does not equal the number of studies due to experimental designs including multiple models. AVM: arteriovenous malformation; CCM: cerebral cavernous malformation.

One author (M.Z.) screened the abstracts and subsequently reviewed the full-text versions of the potentially eligible studies. After screening 2876 publications, we identified 612 full-texts, of which 215 publications were from research on stroke (with 176 ischemic and 42 hemorrhagic records), 52 on infection, 46 on drugs and environmental toxins, 44 each on brain hypoxia and inflammation, and 43 on dementia. All other disorders and stress models retrieved below 30 records **(Figure 2)**. This suggests that EC death is an important feature in studies of the pathophysiology of different brain diseases (Supplemental Figure 1). Among all brain diseases in which EC death has been investigated, we have focused on stroke, as this was by far the most frequently studied and the most prevalent disease.

From publications in stroke, we excluded 63 further publications (64 records) after screening the full-texts, resulting in 152 publications (154 records) to be included **(Figure 2**, Supplemental Tables 1-2). To assess the potential risk of bias in the included studies, we performed a risk of bias analysis according to the Cochrane guidelines [65], with modifications for preclinical research [66], considering the following: i) randomization, ii) sample size calculation, iii) allocation concealment (all selection bias), iv) blinding, v) exposure classification (i.e. target engagement, verification of compound) (both performance/detection bias), vi) complete outcome analysis (attrition bias), vii) selective reporting (reporting bias), viii) conflict of interest, and ix) correct statistical analysis (both other bias) (Supplemental Table 3). The assessment was performed with respect to the endothelial cell death measurements. According to the Cochrane guidelines, each study was judged as ‘low risk', ‘high risk' or as ‘unclear risk', with the last category indicating either the lack of information or uncertainty over the potential for bias [65].

We analyzed diverse *in vitro* and *in vivo* models for both ischemic and hemorrhagic stroke **(Table 1)**, that had a clear focus on oxygen-glucose deprivation (OGD, 109 included publications, 70.8%), while only 39 records (25.3%) used *in vivo* models, and no data from patients was available. 18.8% of studies investigated hemorrhagic stroke (29 records).

**TABLE 1.** Distribution of records according to experimental models used.

**Stroke Subtype**	**Model**	***In vitro*/*in vivo***	**Number of records**
**Ischemic**	Oxygen-glucose deprivation	*in vitro*	109
	Transient middle cerebral artery occlusion	*in vivo*	17
	Permanent middle cerebral artery occlusion	*in vivo*	5
	Embolic stroke	*in vivo*	4
	Glutamate toxicity	*in vitro*	2
	Barrel cortex stroke	*in vivo*	1
	**Total**		**139**
**Hemorrhagic**	Subarachnoid hemorrhage	*in vivo*	10
	Bilirubin toxicity	*in vitro*	5
	Hemin toxicity	*in vitro*	5
	Collagenase toxicity	*in vitro*	2
	Hemoglobin toxicity	*in vitro*	2
	Thrombin toxicity	*in vitro*	2
	Collagenase-induced intracerebral hemorrhage	*in vivo*	1
	Ferrous ammonium sulfate	*in vitro*	1
	Systemic cell-free hemoglobin	*in vivo*	1
	**Total**		**29**

### Time course and location of EC death in stroke

In the studies employing *in vitro* models of stroke, brain EC death was evaluated between 30 min and 48 hours after starting the noxious stimuli that mimic brain ischemia. Rakkar and colleagues exposed human brain microvascular ECs to OGD and found an increase in intracellular calcium, active caspase-3, and TUNEL staining as early as 30 min after OGD onset [67]. Other studies reported reduced cell viability (as detected by MTT) in primary rat brain microvascular ECs [68] and the mouse brain EC line bEnd.3 [69] as early as 2 hours after the onset of OGD.

However, two studies showed a reduction of viability (as detected by MTT) starting only at 6 hours of OGD in primary rat brain microvascular ECs [70, 71] and another study using bEnd.3 cells found decreased cell viability (as detected by MTT) and increased annexin A5/PI staining by 6 and 12 hours of OGD [72]. Other studies in primary mouse brain microvascular ECs and bEnd.3 cells detected the start of cell death (according to MTT and active caspase-3) even later, at 12 hours [73–75] or 16 hours after OGD onset [76].

*In vivo*, in a model of permanent barrel cortex ischemia, Li and colleagues demonstrated that the total number of collagen IV-positive vessels (strictly speaking a marker of the basement membrane and not the ECs) decreased significantly starting at 12 hours after ischemia with a continuous decline up to 72 hours [77]. Friedrich and colleagues assessed cell death in a rat model of subarachnoid hemorrhage (SAH). Of all cells positive for cleaved-caspase-3, ~10% colocalized with collagen IV, which was found in the caudate-putamen, thalamus, and hippocampus as early as 10 min after the injury with a further increase until 24 hours [78]. At 3 days after SAH in rats, Cui and colleagues detected EC death in the anterior and middle cerebral arteries, where about one third of all EC was TUNEL-positive [79]. Similarly, morphological features of EC death were present between 1 and 3 days after transient middle cerebral artery occlusion, including swollen and deformed EC [80, 81], swollen mitochondria with vague crista, and blurred membranes in ECs and edema in the basement membranes [82], while other ECs were still intact.

At 7 days after 90-min middle cerebral artery occlusion in the mouse, the number of CD31-positive cells was reduced in the core but increased in the penumbra [83]. In accordance with these data, Jiang and colleagues reported the survival of some Glut-1-positive vessels inside the core region even until 7 days after ischemic stroke (permanent middle cerebral artery occlusion plus transient ligation of the bilateral common carotid artery) [84]. Glut-1 is an essential glucose transporter expressed by ECs in the brain [85]. Even at 30 days after 60-min middle cerebral artery occlusion, ultrastructural abnormalities (as detected by electron microscopy) were still observed in the striatum capillary endothelia and edematous ECs with swollen mitochondria were adjacent to healthy ECs in the motor cortex of the ipsilateral hemisphere [86].

Thus, EC death occurs both rapidly and later after the onset of stroke conditions, mainly in the core region, but to a certain extent also in remote areas. Further investigations about the location of EC death are warranted and techniques, such as tissue clearing and light sheet microscopy [87] as well as vascular tree analysis [88], may greatly enhance our understanding of the location of EC death in the macro- and microvasculature.

The fast decline of the endothelium may play a role in the early impairment of neurological functions and may interfere with later recovery. However, further research is needed to determine to which extent vessel regression in the ischemic hemisphere is caused by EC death and which consequences result from brain EC death after stroke.

### EC death mechanisms in stroke

Among the different EC cell death mechanisms in stroke identified in this systematic review, apoptosis was the most widely investigated (92 records), followed by autophagy (20 records), while other, more recently defined mechanisms received less attention, such as lysosome-dependent cell death (2 records) and necroptosis (2 records). We found no records dealing with anoikis or ferroptosis (**Table 2**, Supplemental Table 1). Forty-nine publications did not use specific cell death markers. Only 54 out of 152 studies (35.5%) used at least two markers or criteria to confirm the cell death mechanisms as is recommended by the Nomenclature Committee on Cell Death [27].

**TABLE 2.** Distribution of records according to cell death mechanisms and markers.

**Cell death mechanism**	**Marker**	**Number of records**
**Apoptosis**	Cleaved caspases/caspase activity	51
	Annexin A5/propidium iodide or 7-ADD	34
	TUNEL	30
	BCL2 family members (BCL2, BCL-X_L_, BAX, BAK, BAD, BIM, BID)	26
	Mitochondrial depolarization	5
	Phospho-JNK	5
	Cytochrome c release	3
	Apoptotic bodies (EM)	2
	JNK inhibitor	2
	Swollen mitochondria (EM)	2
	Caspase inhibitors	1
	Cytoplasmic DNA fragments	1
	ISL assay for DNA fragmentation	1
	Phosphatidyl serine exposure	1
	Phospho-p38	1
	Shrunken cell body, condensed cytoplasm (EM)	1
	**Total**	**166**
**Autophagy**	LC3-II/I	11
	Autophagy inducers/inhibitors	6
	Beclin-1	6
	DRP1/mitochondrial fission	3
	Formation of autophagosomes (EM)	2
	Mechanistic target of rapamycin (mTOR)	2
	Atg7	1
	BNIP3	1
	Monodansycadaverine	1
	**Total**	**33**
**Lysosome-dependent cell death**	Calpain	1
	Cathepsin	1
	**Total**	**2**
**Necroptosis**	RIP1/RIP3	2
	Necroptosis inhibitors	1
	**Total**	**3**
**Non-specific**	MTT/MTS/WST-1/WST-8/CCK-8	77
	Lactate dehydrogenase (LDH)	43
	Dapi/Hoechst/Trypan blue	18
	Propidium iodide	15
	Microvascular density (CD31/collagen IV/RECA-1)	7
	Intracellular calcium increase	4
	Swollen nucleus, cytoplasm or whole cell (EM)	4
	Acridin Orange/Ethidium bromide	3
	Cellular morphology (phase contrast)	2
	Live/dead staining	2
	Annexin A5 alone	1
	Cell swelling	1
	**Total**	**177**

7-ADD: *7*-aminoactinomycin D; BAD: BCL2 associated agonist of cell death; BAK: BCL2 antagonist/killer 1; BAX: BCL2 associated X, apoptosis regulator; BCL2: apoptosis regulator family; BCL-X_L_: BCL2 like 1 (BCL2L1); BID: BH3 interacting domain death agonist; BIM: BCL2-interacting mediator of cell death (also BCL2 like 11, BCL2L11); BNIP3: BCL2 interacting protein 3; CCK-8: Cell Counting Kit-8; DRP1: dynamin-related protein 1; EM: electron microscopy; ISL: *in situ* ligation; LC3: microtubule-associated protein 1A/1B-light chain 3; MTT: 3-(4,5-dimethylthiazol-2-yl)-2,5-diphenyltetrazolium bromide; phospho-p38: phosphorylated p38 mitogen-activated protein kinase; phospho-JNK: phosphorylated c-Jun N-terminal kinase; RIP: receptor-interacting serine/threonine-protein kinase; TUNEL: TdT-mediated dUTP-biotin nick end labeling; WST: water-soluble tetrazolium.

### Ischemic stroke

Ischemic stroke is the most common subtype of stroke and occurs when the blood supply to an area of the brain is decreased or interrupted, leading to damage of brain cells due to the lack of oxygen and nutrients. Ischemic stroke can be modeled *in vitro* by using OGD, which mimics the decreased supply of oxygen and glucose to cells. *In vivo* ischemic stroke is induced most commonly by permanent, transient middle cerebral artery occlusion and embolic stroke (for review see [89]).

Evidence for apoptotic cell death of ECs comes from a number of investigations. In a detailed study, Hou and colleagues demonstrated that rat primary cerebral microvascular ECs exposed to 8-hour transient OGD presented mitochondrial membrane depolarization, BAD activation, and caspase-1/3 cleavage at 6 hours accompanied by a positive TUNEL staining and externalized phosphatidylserine at 24 hours [90]. In bEnd.3 cells, Liu and colleagues found a time-dependent increase in the levels of cleaved caspases-3 and -9 between 6 and 12 hours of OGD [91]. Other studies corroborate these findings of apoptotic cell death in bEnd.3 cells [92–96], in a human brain microvascular EC line (HBMEC [97–99]) as well as in primary brain microvascular ECs from rats [100, 101] and mice [75, 102–104].

For the assessment of transient cerebral ischemia, OGD is followed by a phase of reoxygenation in normal culture medium. Whether transient ischemia causes additional damage to ECs remains unclear so far. One study noticed more caspase-3/7-positive HBMECs in transient than in permanent OGD [105], while another study in the same cell line showed a non-significant reduction in the levels of caspase-3/7 in transient compared to permanent OGD. TUNEL staining led to comparable results under both conditions [67].

Besides apoptosis, other cell death mechanisms have been investigated in OGD. When exposed to transient OGD of up to 16 hours, EC death is modulated by autophagy (as detected by an increase in LC3-II or the autophagy-specific pharmacological modulation of EC survival) [106–109]. In a detailed mechanistic study investigating the protein levels of BCL2, BAX, and cleaved caspase-3, Chen and colleagues found no evidence that brain microvascular ECs (BMEC) undergo apoptotic cell death when exposed to 2 hours of OGD. In turn, they reported elevated protein levels of DRP1 and phospho-DRP1, while they strikingly observed a decrease in LC3-II. Treatment with the autophagy inducer rapamycin significantly improved cell viability (as detected by MTT) while the autophagy inhibitors 3-methyladenine and chloroquine decreased it. Electron microscopy revealed tubular and damaged mitochondria inside autophagosomes [106]. These data would fit to the concept of a protective autophagy eliminating damaged mitochondria, a process that seems to be based on DRP1 instead of LC3 [110].

Endothelial autophagy is not restricted to *in vitro* paradigms. Li and colleagues found elevated beclin-1 expression in cerebral microvessels between 6 and 24 hours after the onset of focal cerebral ischemia [77]. Garbuzova-Davis and colleagues observed the formation of autophagosomes (as detected by electron microscopy and beclin-1 immunostaining) in ECs of both the ipsilateral and contralateral striatum and the ipsilateral motor cortex 30 days after 60-min middle cerebral artery occlusion in the rat [86].

In contrast, a study by Engelhardt and colleagues investigated primary rat brain EC exposed to 24 and 48 hours of oxygen deprivation (OD) or permanent OGD. Only OGD but not OD alone reduced cell viability (as detected by MTT), which was more pronounced after 48 hours. They further investigated apoptotic cell death and autophagy in a rat brain endothelial cell line (RBE4). Despite an initial increase in BCL2 interacting protein 3 (*Bnip3)* mRNA, BNIP3 protein was decreased after 48 hours of OGD [111]. Notably, the function of BNIP3 is still controversial. It is involved in the apoptotic process, but also seems to contribute to the cell survival via autophagy [112, 113]. In the same study by Engelhardt and colleagues, a reduction of the apoptotic marker BAX as well as the autophagy markers beclin-1 and LC3-II was detected in ECs upon OGD exposure [111]. However, before concluding from these data that OGD does not induce autophagy in ECs, it has to be taken into account that changes were only observed at 48 hours, a time point when the survival of the cells was severely reduced and those cells that did survive may, for some reason, have been more resistant to the stress.

Interestingly, Wang and colleagues knocked out autophagy related 7 (*Atg7*) specifically in ECs and subjected these mice to 60 min of middle cerebral artery occlusion. They found that endothelial knockout of *Atg7* significantly reduced infarct sizes, neurological outcome, and inflammatory responses. They further reproduced the anti-inflammatory effect by using shAtg7 in HBMECs subjected to transient OGD, where shAtg7 reduced IKKβ phosphorylation, leading to less NF-κB activation and a lower transcription of pro-inflammatory cytokines, which they propose to be independent of the role of ATG7 in autophagy [114]. Thus, the effect of *Atg7* deficiency may not provide evidence that endothelial autophagy plays an important role in cerebral ischemia.

For the other cell death mechanisms, there are only very few reports in the literature, which may reflect the notion that these cell death mechanisms do not occur. More likely, however, they have not been investigated in much detail so far. For example, for necroptotic cell death signaling, Luo and colleagues found that the RIPK1 inhibitor necrostatin-1 abrogated the increase in the number of PI-positive bEnd.3 cells and the levels of RIPK1 protein after 6 hours of OGD, while caspase activity remained unchanged with and without necrostatin-1 exposure after OGD [69]. However, only the total protein levels of RIPK1 were shown and an increase in this kinase does not conclusively indicate enhanced necroptosis (see section **Detection of EC death)**.

Two *in vivo* studies can be interpreted as evidence for a lysosome-dependent cell death of ECs during cerebral ischemia. ElAli and Hermann reported that transient middle cerebral artery occlusion activated the protease calpain indicating lysosomal damage. The parallel activation of caspase-3 in this study may represent a sign of apoptotic cell death [115]. Another study that induced cerebral ischemia in rats by microsphere embolism [116] observed cathepsin activity as a marker of lysosome-dependent cell death in vessels in combination with autophagy (increase in LC3-II). Unfortunately, most of these studies focused on a specific subset of cell death mechanisms, which makes it difficult to judge the relative importance of the various cell death routines at present.

### Hemorrhagic stroke

Although less common than its ischemic counterpart, hemorrhagic stroke remains a major cause of mortality and permanent disability worldwide. It has both primary (physical disruption, increased intracranial pressure) and secondary (clot-derived cytotoxic factors, neuroinflammation, edema, secondary ischemia) injury components. Increasing evidence suggests that hemoglobin is one of the vital factors contributing to the cell autonomous and non-cell autonomous injury [117], which may lead to cell death mechanisms that differ from those occurring in ischemic stroke.

Brain hemorrhage can be modeled *in vitro* by using blood breakdown products, such as hemoglobin, heme or its oxidized form hemin, bilirubin, thrombin, or iron. In an extensive study, Sukumari-Ramesh and colleagues investigated hemin toxicity in mouse bEnd.3 and human brain microvascular cells after 6, 12, or 18 hours of treatment [118]. They found a concentration- and time-dependent decrease in cellular viability (as detected by MTT) in both cell types. By challenging bEnd.3 cells with hemin for 18 hours, they further observed that the cells were positive for annexin A5. In addition, hemin treatment increased the levels of cleaved caspase-3 in bEnd.3 cells, an effect that was reversed by incubation with the pan-caspase inhibitor Z-VAD-fmk that also prevented hemin-induced toxicity suggesting that the cells die by apoptosis. Notably, hemin toxicity was abrogated by ascorbic acid (vitamin C), trolox (vitamin E analog), and deferoxamine (iron chelator and hypoxia-inducible factor prolyl hydroxylase inhibitor). As trolox and deferoxamine have been suggested to belong to a set of non-specific pharmacological inhibitors of ferroptosis [51, 119], it can thus be speculated that ferroptosis is another potential cell death mechanism in ECs upon blood exposure.

These data suggest that there may also be a mixture of multiple cell death pathways involved in EC demise after brain hemorrhage, i.e. ferroptosis (trolox and deferoxamine) and apoptosis (cleaved caspase-3, annexin A5), which is not unlikely as the coexistence of multiple cell death subroutines (ferroptosis, necroptosis, and likely autophagy) has been shown in neurons already [51, 120]. Nevertheless, these findings warrant further molecular investigation, especially regarding the involvement of the more recently defined ferroptotic cell death.

Unfortunately, the data on the role of other blood breakdown products in brain EC death is still limited. For example, Liu and colleagues investigated cell death of human brain vascular ECs exposed to heme for 24 hours and found a concentration-dependent decrease in cell viability (as detected by MTT) and an increase in TUNEL reactivity [121]. By applying bilirubin for 24 hours in bEnd.3 cells, Kapitulnik and colleagues demonstrated a concentration-dependent increase in the caspase activity and a decrease in the cell numbers, which were further exacerbated under hyperglycemic conditions [122]. This was recapitulated in primary rat brain microvascular ECs that also showed apoptotic bodies in another study [123].

Most *in vivo* studies in hemorrhagic stroke only assessed apoptotic cell death. This form of cell death was found in ECs of the caudate-putamen, thalamus, and hippocampus [78], in the anterior and middle cerebral artery [79] and in microvessels [124, 125] after SAH in the rat as well as in the perihematomal area in a collagenase model of intracerebral hemorrhage [126]. While Yan and colleagues also detected apoptotic markers (i.e. levels of BAX, BAK, and TUNEL) in EC death of the microvasculature in the hippocampus after SAH, they further reported an increase in the autophagy marker DRP1 [127].

Together, hemorrhagic stroke leads to EC death, just like ischemic stroke, but the underlying mechanisms seem to be different and may include ferroptosis in addition to apoptosis.

### Differential vulnerability of brain cells after stroke

As stroke affects multiple components of the neurovascular unit, we asked whether the individual cell types are differentially affected, i.e. more vulnerable and/or die in sequence or simultaneously after stroke.

Lyden and colleagues reported that transient OGD of 2 hours was required to kill 80% of primary neurons in culture, 6 hours for the same death rate of primary ECs, and 10 hours for primary astrocytes (as detected by MTT or LDH) after 24 hours [128]. Similarly, Li and colleagues reported that 1 hour of OGD in primary rat cortical neurons was sufficient to induce 50% cell death after 24 hours, while the EC line bEnd.3 required 3 hours of OGD (as detected by MTT, LDH, and active caspase-3) [129]. In another study, Redzic and colleagues identified that primary rat ECs were more susceptible to OGD than primary rat astrocytes, pericytes, and microglia: While ECs started to die already after 1 hour, astrocytes and pericytes were resistant up to 2 hours and microglia up to 4 hours of OGD (as detected by LDH) [130].

In contrast, in permanent OGD, Engelhardt and colleagues showed that primary rat brain ECs, pericytes and astrocytes were similarly affected by 24 and 48 hours of OGD, while only ECs and astrocytes lost half of their metabolic activity (as detected by MTT) when glucose was removed under normoxic conditions. However, in the same study, the autophagy marker LC3-II decreased in ECs upon OGD exposure indicating autophagy inhibition, whereas the levels of LC3-II increased in astrocytes and pericytes suggestive of induced autophagy in these cell types [111]. Similarly, Huang and colleagues reported neurons, astrocytes, and ECs to be similarly affected after 6, 12, 24, and 48 hours of OGD (as detected by MTT), with a common protective mechanism via increasing vascular endothelial growth factor expression [131].

*In vivo*, Zuo and colleagues reported that 10% of all ECs but 40% of all neurons were TUNEL-positive at 3 days after 2-hour middle cerebral artery occlusion in rats [132]. However, the analysis was confined to only one time point after stroke. To address this issue, Jiang and colleagues performed an extensive time course analysis of the survival of the different cell types in the ischemic core after permanent embolic stroke: more than one third of the neurons died within the first 6 hours and reached a death rate of almost 100% at 7 days (according to TUNEL staining) with few exceptions (as detected by electron microscopy). Interestingly, the authors did not detect extensive EC death within the first 2 days after stroke, but the number of Glut-1-positive vessels decreased at 3 and 7 days [84]. In addition, the authors showed that the overall number of non-neuronal cells remained stable up to 3 days and dramatically increased at 7 days, which was related to increasing numbers of astrocytes and microglia [84].

Since most experiments showed an earlier onset of cell death for neurons than for ECs, it may be speculated that brain EC death is a secondary effect of parenchymal injury. However, this hypothesis needs to be further investigated.

Differential overall vulnerability may also implicate different underlying cell death mechanisms and consequently varying therapeutic effects. Indeed, Lyden and colleagues detected differential effects of hypothermia protection on primary neurons, ECs, and astrocytes in culture. They found that shorter hypothermia was superior to longer hypothermia *in vivo*, which they related to the suppression of astrocytic paracrine support of neurons during injury [128]. In another study, Li and colleagues compared the time course of neuronal and EC autophagy (as detected by beclin-1) that eventually led to cell death (according to TUNEL staining) and detected a simultaneous increase in the death of both cell types between 6 and 24 hours after murine barrel cortex stroke, which lasted until 3 days only in neurons [77].

However, a systematic characterization of the cell death mechanisms after stroke in the different cell types side-by-side still needs to be performed.

## EC DEATH AND THE NO-REFLOW PHENOMENON – WHY SMALL VESSELS MATTER

A decisive factor for the cellular demise in ischemic stroke is the restoration of blood flow upon occlusion. However, the recanalization rates after recombinant tissue-type plasminogen activator thrombolysis in clinical trials can vary and failure can be up to 30-40% [133]. This failure may be due to vessel loss or ongoing occlusion. In myocardial ischemia, the no-reflow phenomenon (the inability to reperfuse despite the removal of the large vessel occlusion) is related to microvascular obstruction [134]. The consequences of blood vessel occlusion and associated EC death after stroke likely also depend on the vessel type that is affected, considering the differences between larger vessels and smaller vessels such as capillaries. It has been estimated that capillaries in the rat cortex contain only 2.3 ECs on average between two branch points [135]. In contrast, several ECs form the wall of larger vessels, including arterioles and venules.

It is therefore plausible that the loss of single cells is more difficult to compensate in small vessels. Morphological changes associated with apoptosis have been demonstrated to block the lumen of the microvessels in the periphery [136]. Hence, endothelial apoptosis may lead to the shut-off of blood flow in the damaged capillary and reduce its leakage, which would reduce the exposure of ECs to blood breakdown products. In accordance with this notion, the death of microvascular ECs does not always lead to intracerebral hemorrhage [15]. Notably, string vessels often involve a full capillary branch implying that more than one EC are involved [16]. Upon the apoptosis of an initiator EC, the loss of perfusion and the reduction of shear stress may subsequently interfere with the viability of adjacent ECs in the same capillary because shear stress, especially laminar shear stress protects ECs against noxious stimuli [137]. Alternatively, cell death may spread by a mechanism such as apoptosis-induced apoptosis [138].

Considering that relatively large components of the blood need to pass through narrow capillaries (~3-5 μm in diameter), it is not surprising that capillaries are obstructed transiently, even in healthy animals [139, 140]. Erdener and colleagues estimated that 7.5% of capillaries experience a stall over 9 min of recording of erythrocyte flux in the healthy mouse brain *in vivo* [141]. Occluding emboli can be extruded into the vessel lumen or through the endothelial cell by a process termed angiopathy [142, 143], restoring blood flow.

Further work demonstrated that the failure to recanalize within 20 min leads to a progressive pruning of about 30% of capillaries, leaving the remaining segment connected to the adjacent, flowing capillary (string vessels). Vessel segments retract, which was associated with an increase in EC nuclei around pruned branch points [143], suggestive of EC regression and integration into the adjacent capillaries similar to that found during development [11, 144].

Further research needs to address the precise role of EC death in the causal or consequential events leading to the vessel loss and no-reflow phenomenon considering the differences in larger and smaller vessels.

## EC DEATH AND BBB DISRUPTION

EC death is often associated with a disruption of the BBB. Krueger and colleagues proposed that EC loss causes BBB breakdown [145], as by using electron microscopy they observed a loss of endothelial layer integrity in brain areas with FITC-albumin extravasation into the neuropil.

However, whether the loss of ECs creates holes in the vessel wall before the blood flow in the damaged capillaries stops is still unclear. One indication comes from a study using brain endothelial *Nemo*-deficient mice. NEMO is an essential part of the classical NF-κB signaling pathway and, expressed in endothelial cells, protects the neurovascular unit from injury. In mice with *Nemo*-deficient ECs, distinct signaling pathways mediated the loss of capillaries and the disruption of the BBB [15]. This suggests that BBB leakage is not always due to the execution of cell death but rather reflects preceding damage of vessels.

In larger vessels, morphological changes associated with EC death are less likely to shut-off blood flow. EC death and subsequent holes in the vessel wall may create leakage and lead to hemorrhages. In support of this notion, toxin-induced EC death was accompanied by hemorrhages in various tissues [146–148]. EC death may be the mechanism by which Dengue virus infection can trigger brain hemorrhages [149]. In zebrafish, deletion of cIap1, an inhibitor of apoptosis, triggered EC apoptosis and hemorrhage in the brain and other organs [150]. These data indicate that EC death may be an important cause of intracranial hemorrhage.

## LIMITATIONS OF THE SYSTEMATIC REVIEW

Our analysis has several limitations:

i) Only one database (PUBMED) was screened and only one person screened and extracted the data (M.Z.).

ii) The majority of the studies used the OGD model (70.8%). This is reasonable due to availability and the ease to investigate EC cell death *in vitro* specifically. While the results with respect to the time course, differential vulnerability, and cell death subroutine were similar in the reported *in vitro* and *in vivo* studies, it cannot be fully excluded that differences occur. In addition, the length and severity of ischemia or hemorrhage likely also determine the time course and type of EC death. However, at present, there are not enough data to draw any firm conclusions.

iii) The overall reporting of quality according to the Stroke Therapy Academic Industry Roundtable [151] was mostly incomplete (numbers are with respect to EC death assessment experiments of the included studies): Only 27 out of 152 studies reported randomization, one study reported *a priori* sample size calculation, 17 studies allocation concealment, 23 studies blinding of researchers, two studies exposure classification (i.e. target validation, verification of compound), and 114 studies provided a conflict of interest statement (Supplemental Table 3). We were able to rule out attrition bias only for one study, while four studies were clearly biased for incomplete outcome analysis due to unexplained unequal numbers of biological replicates. Selective reporting was ruled out only for two studies. Common statistical issues identified are the use of statistical tests that require normally distributed data (e.g., t-test or ANOVA) without reporting that normal distribution or the homogeneity of variance was tested or confirmed [66] and a low sample size that is insufficient (n=3-4 per group) to assume normal distribution. In this systematic review, incorrect statistical tests (parametric tests when the sample size was insufficient or t-tests for multiple comparisons) were reported in 87 studies.

## CONCLUSIONS

EC death occurs both rapidly and at later time points after the onset of stroke, which may play a role in the early impairment of neurological functions and the later difficulties during recovery. The mechanisms of cell death of brain ECs are complex and include multiple cell death subroutines, including apoptosis, autophagy, necroptosis, and maybe ferroptosis. Further characterization should include at least two independent markers/criteria for each cell death subroutine according to the recommendations of the Nomenclature Committee on Cell Death. This will give rise to a comprehensive understanding of the cell death signaling, which will pave the way to new therapeutic targets and approaches for the treatment of patients suffering from stroke and potentially other cerebrovascular diseases.

## DATA AVAILABILITY

All full-texts screened for the systematic review including PMIDs are available in the supplemental data (Supplemental Table 1 and 2). A file of all abstracts included by the search strategy (as detailed in the Supplemental Data) can be obtained upon request from the authors.

## SUPPLEMENTAL MATERIAL

Click here for supplemental data file.

All supplemental data for this article are also available online at http://www.cell-stress.com/researcharticles/2019a-zille-cell-stress/.

## AUTHOR CONTRIBUTION

M.Z. and M.S. designed the research. M.Z. performed the screening of the studies and extracted the data. J.L. provided Figure 1. M.Z. and M.S. wrote the manuscript. All authors discussed the manuscript and approved the final version.

## Abbreviatons:

ASM: – acid sphingomyelinase,
ATG: – autophagy related,
BBB: – blood brain barrier,
EC: – endothelial cell,
HBMEC: – human brain microvascular EC,
LDH: – lactate dehydrogenase,
MTT: – 3-(4,5-Dimethylthiazol-2-yl)-2,5-diphenyltetrazolium bromide,
OD: – oxygen deprivation,
OGD: – oxygen-glucose deprivation,
PI: – propidium iodide,
SAH: – subarachnoid hemorrhage,
TdT: – terminal deoxynucleotidyl transferase,
TUNEL: – TdT-mediated dUTP nick end labeling.

## References

[1] Clarke DD, Sokoloff L, Siegel GJ, Agranoff BW, Albers RW, Fisher SK, Uhler MD (1999). Circulation and energy metabolism of the brain.. Basic Neurochemistry: Molecular, Cellular, and Medical Aspects..

[2] Schlageter KE, Molnar P, Lapin GD, Groothuis DR (1999). Microvessel organization and structure in experimental brain tumors: microvessel populations with distinctive structural and functional properties.. Microvasc Res.

[3] Banks WA, Kovac A, Morofuji Y (2018). Neurovascular unit crosstalk: Pericytes and astrocytes modify cytokine secretion patterns of brain endothelial cells.. J Cereb Blood Flow Metab.

[4] Mancuso MR, Kuhnert F, Kuo CJ (2008). Developmental angiogenesis of the central nervous system.. Lymphat Res Biol.

[5] Plein A, Fantin A, Denti L, Pollard JW, Ruhrberg C (2018). Erythro-myeloid progenitors contribute endothelial cells to blood vessels.. Nature.

[6] Daneman R, Zhou L, Agalliu D, Cahoy JD, Kaushal A, Barres BA (2010). The mouse blood-brain barrier transcriptome: a new resource for understanding the development and function of brain endothelial cells.. PLoS ONE.

[7] Chu C, Li JY, Boado RJ, Pardridge WM (2008). Blood-brain barrier genomics and cloning of a novel organic anion transporter.. J Cereb Blood Flow Metab.

[8] Li JY, Boado RJ, Pardridge WM (2002). Rat blood-brain barrier genomics. II.. J Cereb Blood Flow Metab.

[9] Li JY, Boado RJ, Pardridge WM (2001). Blood-brain barrier genomics.. J Cereb Blood Flow Metab.

[10] Helms HC, Abbott NJ, Burek M, Cecchelli R, Couraud PO, Deli MA, Forster C, Galla HJ, Romero IA, Shusta EV, Stebbins MJ, Vandenhaute E, Weksler B, Brodin B (2016). In vitro models of the blood-brain barrier: An overview of commonly used brain endothelial cell culture models and guidelines for their use.. J Cereb Blood Flow Metab.

[11] Chen Q, Jiang L, Li C, Hu D, Bu JW, Cai D, Du JL (2012). Haemodynamics-driven developmental pruning of brain vasculature in zebrafish.. PLoS Biol.

[12] Harb R, Whiteus C, Freitas C, Grutzendler J (2013). In vivo imaging of cerebral microvascular plasticity from birth to death.. J Cereb Blood Flow Metab.

[13] Zhang Y, Xu B, Chen Q, Yan Y, Du J, Du X (2018). Apoptosis of Endothelial Cells Contributes to Brain Vessel Pruning of Zebrafish During Development.. Front Mol Neurosci.

[14] Korn C, Augustin HG (2015). Mechanisms of Vessel Pruning and Regression.. Dev Cell.

[15] Ridder DA, Wenzel J, Muller K, Tollner K, Tong XK, Assmann JC, Stroobants S, Weber T, Niturad C, Fischer L, Lembrich B, Wolburg H, Grand'Maison M, Papadopoulos P, Korpos E, Truchetet F, Rades D, Sorokin LM, Schmidt-Supprian M, Bedell BJ, Pasparakis M, Balschun D, D'Hooge R, Loscher W, Hamel E, Schwaninger M (2015). Brain endothelial TAK1 and NEMO safeguard the neurovascular unit.. J Exp Med.

[16] Brown WR (2010). A review of string vessels or collapsed, empty basement membrane tubes.. J Alzheimers Dis.

[17] Errede M, Mangieri D, Longo G, Girolamo F, de Trizio I, Vimercati A, Serio G, Frei K, Perris R, Virgintino D (2018). Tunneling nanotubes evoke pericyte/endothelial communication during normal and tumoral angiogenesis.. Fluids Barriers CNS.

[18] Brown WR, Thore CR (2011). Review: cerebral microvascular pathology in ageing and neurodegeneration.. Neuropathol Appl Neurobiol.

[19] Park MH, Lee JY, Park KH, Jung IK, Kim KT, Lee YS, Ryu HH, Jeong Y, Kang M, Schwaninger M, Gulbins E, Reichel M, Kornhuber J, Yamaguchi T, Kim HJ, Kim SH, Schuchman EH, Jin HK, Bae JS (2018). Vascular and Neurogenic Rejuvenation in Aging Mice by Modulation of ASM.. Neuron.

[20] Schwartz LM (1991). The role of cell death genes during development.. Bioessays.

[21] Kerr JF, Wyllie AH, Currie AR (1972). Apoptosis: a basic biological phenomenon with wide-ranging implications in tissue kinetics.. Br J Cancer.

[22] Galluzzi L, Aaronson SA, Abrams J, Alnemri ES, Andrews DW, Baehrecke EH, Bazan NG, Blagosklonny MV, Blomgren K, Borner C, Bredesen DE, Brenner C, Castedo M, Cidlowski JA, Ciechanover A, Cohen GM, De Laurenzi V, De Maria R, Deshmukh M, Dynlacht BD, El-Deiry WS, Flavell RA, Fulda S, Garrido C, Golstein P, Gougeon ML, Green DR, Gronemeyer H, Hajnoczky G, Hardwick JM (2009). Guidelines for the use and interpretation of assays for monitoring cell death in higher eukaryotes.. Cell Death Differ.

[23] Degterev A, Huang Z, Boyce M, Li Y, Jagtap P, Mizushima N, Cuny GD, Mitchison TJ, Moskowitz MA, Yuan J (2005). Chemical inhibitor of nonapoptotic cell death with therapeutic potential for ischemic brain injury.. Nat Chem Biol.

[24] Chautan M, Chazal G, Cecconi F, Gruss P, Golstein P (1999). Interdigital cell death can occur through a necrotic and caspase-independent pathway.. Curr Biol.

[25] Galluzzi L, Vitale I, Aaronson SA, Abrams JM, Adam D, Agostinis P, Alnemri ES, Altucci L, Amelio I, Andrews DW, Annicchiarico-Petruzzelli M, Antonov AV, Arama E, Baehrecke EH, Barlev NA, Bazan NG, Bernassola F, Bertrand MJM, Bianchi K, Blagosklonny MV, Blomgren K, Borner C, Boya P, Brenner C, Campanella M, Candi E, Carmona-Gutierrez D, Cecconi F, Chan FK, Chandel NS (2018). Molecular mechanisms of cell death: recommendations of the Nomenclature Committee on Cell Death 2018.. Cell Death Differ.

[26] Zille M, Farr TD, Przesdzing I, Muller J, Sommer C, Dirnagl U, Wunder A (2012). Visualizing cell death in experimental focal cerebral ischemia: promises, problems, and perspectives.. J Cereb Blood Flow Metab.

[27] Galluzzi L, Vitale I, Abrams JM, Alnemri ES, Baehrecke EH, Blagosklonny MV, Dawson TM, Dawson VL, El-Deiry WS, Fulda S, Gottlieb E, Green DR, Hengartner MO, Kepp O, Knight RA, Kumar S, Lipton SA, Lu X, Madeo F, Malorni W, Mehlen P, Nunez G, Peter ME, Piacentini M, Rubinsztein DC, Shi Y, Simon HU, Vandenabeele P, White E, Yuan J (2012). Molecular definitions of cell death subroutines: recommendations of the Nomenclature Committee on Cell Death 2012.. Cell Death Differ.

[28] Gavrieli Y, Sherman Y, Ben-Sasson SA (1992). Identification of programmed cell death in situ via specific labeling of nuclear DNA fragmentation.. J Cell Biol.

[29] Dmitrieva NI, Burg MB (2007). Osmotic stress and DNA damage.. Methods Enzymol.

[30] Cohen GM, Sun XM, Snowden RT, Dinsdale D, Skilleter DN (1992). Key morphological features of apoptosis may occur in the absence of internucleosomal DNA fragmentation.. Biochem J.

[31] Charriaut-Marlangue C, Ben-Ari Y (1995). A cautionary note on the use of the TUNEL stain to determine apoptosis.. Neuroreport.

[32] Grasl-Kraupp B, Ruttkay-Nedecky B, Koudelka H, Bukowska K, Bursch W, Schulte-Hermann R (1995). In situ detection of fragmented DNA (TUNEL assay) fails to discriminate among apoptosis, necrosis, and autolytic cell death: a cautionary note.. Hepatology.

[33] de Torres C, Munell F, Ferrer I, Reventos J, Macaya A (1997). Identification of necrotic cell death by the TUNEL assay in the hypoxic-ischemic neonatal rat brain.. Neurosci Lett.

[34] Galluzzi L, Joza N, Tasdemir E, Maiuri MC, Hengartner M, Abrams JM, Tavernarakis N, Penninger J, Madeo F, Kroemer G (2008). No death without life: vital functions of apoptotic effectors.. Cell Death Differ.

[35] Segawa K, Nagata S (2015). An Apoptotic 'Eat Me' Signal: Phosphatidylserine Exposure.. Trends Cell Biol.

[36] Ran S, Thorpe PE (2002). Phosphatidylserine is a marker of tumor vasculature and a potential target for cancer imaging and therapy.. Int J Radiat Oncol Biol Phys.

[37] Yang MY, Chuang H, Chen RF, Yang KD (2002). Reversible phosphatidylserine expression on blood granulocytes related to membrane perturbation but not DNA strand breaks.. J Leukoc Biol.

[38] Kenis H, Zandbergen HR, Hofstra L, Petrov AD, Dumont EA, Blankenberg FD, Haider N, Bitsch N, Gijbels M, Verjans JW, Narula N, Narula J, Reutelingsperger CP (2010). Annexin A5 uptake in ischemic myocardium: demonstration of reversible phosphatidylserine externalization and feasibility of radionuclide imaging.. J Nucl Med.

[39] Kroemer G, Galluzzi L, Vandenabeele P, Abrams J, Alnemri ES, Baehrecke EH, Blagosklonny MV, El-Deiry WS, Golstein P, Green DR, Hengartner M, Knight RA, Kumar S, Lipton SA, Malorni W, Nunez G, Peter ME, Tschopp J, Yuan J, Piacentini M, Zhivotovsky B, Melino G, Nomenclature Committee on Cell D (2009). Classification of cell death: recommendations of the Nomenclature Committee on Cell Death 2009.. Cell Death Differ.

[40] Zhang Y, Chen X, Gueydan C, Han J (2018). Plasma membrane changes during programmed cell deaths.. Cell Res.

[41] Strilic B, Yang L, Albarran-Juarez J, Wachsmuth L, Han K, Muller UC, Pasparakis M, Offermanns S (2016). Tumour-cell-induced endothelial cell necroptosis via death receptor 6 promotes metastasis.. Nature.

[42] Haeckel A, Appler F, Figge L, Kratz H, Lukas M, Michel R, Schnorr J, Zille M, Hamm B, Schellenberger E (2014). XTEN-annexin A5: XTEN allows complete expression of long-circulating protein-based imaging probes as recombinant alternative to PEGylation.. J Nucl Med.

[43] Zille M, Harhausen D, De Saint-Hubert M, Michel R, Reutelingsperger CP, Dirnagl U, Wunder A (2014). A dual-labeled Annexin A5 is not suited for SPECT imaging of brain cell death in experimental murine stroke.. J Cereb Blood Flow Metab.

[44] Bahmani P, Schellenberger E, Klohs J, Steinbrink J, Cordell R, Zille M, Muller J, Harhausen D, Hofstra L, Reutelingsperger C, Farr TD, Dirnagl U, Wunder A (2011). Visualization of cell death in mice with focal cerebral ischemia using fluorescent annexin A5, propidium iodide, and TUNEL staining.. J Cereb Blood Flow Metab.

[45] Bestebroer J, V'Kovski P, Mauthe M, Reggiori F (2013). Hidden behind autophagy: the unconventional roles of ATG proteins.. Traffic.

[46] Stockwell BR, Friedmann Angeli JP, Bayir H, Bush AI, Conrad M, Dixon SJ, Fulda S, Gascon S, Hatzios SK, Kagan VE, Noel K, Jiang X, Linkermann A, Murphy ME, Overholtzer M, Oyagi A, Pagnussat GC, Park J, Ran Q, Rosenfeld CS, Salnikow K, Tang D, Torti FM, Torti SV, Toyokuni S, Woerpel KA, Zhang DD (2017). Ferroptosis: A Regulated Cell Death Nexus Linking Metabolism, Redox Biology, and Disease.. Cell.

[47] Christofferson DE, Li Y, Yuan J (2014). Control of life-or-death decisions by RIP1 kinase.. Annu Rev Physiol.

[48] Silke J, Rickard JA, Gerlic M (2015). The diverse role of RIP kinases in necroptosis and inflammation.. Nat Immunol.

[49] Berger SB, Kasparcova V, Hoffman S, Swift B, Dare L, Schaeffer M, Capriotti C, Cook M, Finger J, Hughes-Earle A, Harris PA, Kaiser WJ, Mocarski ES, Bertin J, Gough PJ (2014). Cutting Edge: RIP1 kinase activity is dispensable for normal development but is a key regulator of inflammation in SHARPIN-deficient mice.. J Immunol.

[50] Guo H, Omoto S, Harris PA, Finger JN, Bertin J, Gough PJ, Kaiser WJ, Mocarski ES (2015). Herpes simplex virus suppresses necroptosis in human cells.. Cell Host Microbe.

[51] Zille M, Karuppagounder SS, Chen Y, Gough PJ, Bertin J, Finger J, Milner TA, Jonas EA, Ratan RR (2017). Neuronal Death After Hemorrhagic Stroke In Vitro and In Vivo Shares Features of Ferroptosis and Necroptosis.. Stroke.

[52] Newton K, Wickliffe KE, Maltzman A, Dugger DL, Strasser A, Pham VC, Lill JR, Roose-Girma M, Warming S, Solon M, Ngu H, Webster JD, Dixit VM (2016). RIPK1 inhibits ZBP1-driven necroptosis during development.. Nature.

[53] Webster JD, Solon M, Haller S, Newton K (2018). Detection of Necroptosis by Phospho-RIPK3 Immunohistochemical Labeling.. Methods Mol Biol.

[54] Gong Y-N, Guy C, Olauson H, Becker JU, Yang M, Fitzgerald P, Linkermann A, Green DR (2017). ESCRT-III Acts Downstream of MLKL to Regulate Necroptotic Cell Death and Its Consequences.. Cell.

[55] Degterev A, Hitomi J, Germscheid M, Ch'en IL, Korkina O, Teng X, Abbott D, Cuny GD, Yuan C, Wagner G, Hedrick SM, Gerber SA, Lugovskoy A, Yuan J (2008). Identification of RIP1 kinase as a specific cellular target of necrostatins.. Nat Chem Biol.

[56] Takahashi N, Duprez L, Grootjans S, Cauwels A, Nerinckx W, DuHadaway JB, Goossens V, Roelandt R, Van Hauwermeiren F, Libert C, Declercq W, Callewaert N, Prendergast GC, Degterev A, Yuan J, Vandenabeele P (2012). Necrostatin-1 analogues: critical issues on the specificity, activity and in vivo use in experimental disease models.. Cell Death Dis.

[57] Huang T, Karsy M, Zhuge J, Zhong M, Liu D (2013). B-Raf and the inhibitors: from bench to bedside.. J Hematol Oncol.

[58] Chacon E, Acosta D, Lemasters JL, Castell JV, Gómez-Lechón MJ (1997). Primary Cultures of Cardiac Myocytes as In Vitro Models for Pharmacological and Toxicological Assessments.. In Vitro Methods in Pharmaceutical Research..

[59] Takahashi S, Abe T, Gotoh J, Fukuuchi Y (2002). Substrate-dependence of reduction of MTT: a tetrazolium dye differs in cultured astroglia and neurons.. Neurochem Int.

[60] Koh JY, Choi DW (1987). Quantitative determination of glutamate mediated cortical neuronal injury in cell culture by lactate dehydrogenase efflux assay.. J Neurosci Methods.

[61] Sweeney MD, Kisler K, Montagne A, Toga AW, Zlokovic BV (2018). The role of brain vasculature in neurodegenerative disorders.. Nat Neurosci.

[62] Zlokovic BV (2008). The blood-brain barrier in health and chronic neurodegenerative disorders.. Neuron.

[63] Rizzo MT, Leaver HA (2010). Brain endothelial cell death: modes, signaling pathways, and relevance to neural development, homeostasis, and disease.. Mol Neurobiol.

[64] Moher D, Liberati A, Tetzlaff J, Altman DG, Group P (2009). Preferred reporting items for systematic reviews and meta-analyses: the PRISMA statement.. PLoS Med.

[65] Higgins J, Green S (2011). Cochrane Handbook for Systematic Reviews of Interventions Version 5.1.0 [updated March 2011].. The Cochrane Collaboration, 2011..

[66] Office of Health Assessment and Translation (2015). OHAT Risk of Bias Tool for Human and Animal Studies.. https://ntp.niehs.nih.gov/pubhealth/hat/review/index-2.html.

[67] Rakkar K, Bayraktutan U (2016). Increases in intracellular calcium perturb blood-brain barrier via protein kinase C-alpha and apoptosis.. Biochim Biophys Acta.

[68] Zhang J, Qi C, Yang P, Chen X, Liu Y (2017). Activation of Wnt3alpha/beta-catenin signal pathway attenuates apoptosis of the cerebral microvascular endothelial cells induced by oxygen-glucose deprivation.. Biochem Biophys Res Commun.

[69] Luo S, Li S, Zhu L, Fang SH, Chen JL, Xu QQ, Li HY, Luo NC, Yang C, Luo D, Li L, Ma XH, Zhang R, Wang H, Chen YB, Wang Q (2017). Effect of baicalin on oxygen-glucose deprivation-induced endothelial cell damage.. Neuroreport.

[70] Ji BS, Cen J, He L, Liu M, Liu YQ, Liu L (2013). Modulation of P-glycoprotein in rat brain microvessel endothelial cells under oxygen glucose deprivation.. J Pharm Pharmacol.

[71] Zhang X, Fu C, Chen B, Xu Z, Zeng Z, He L, Lu Y, Chen Z, Liu X (2019). Autophagy Induced by Oxygen-Glucose Deprivation Mediates the Injury to the Neurovascular Unit.. Med Sci Monit.

[72] Xu M, Yang X, Zeng Q, He H, Lu P, Huang G (2017). BIRC5 is a novel target of peroxisome proliferatoractivated receptor gamma in brain microvascular endothelium cells during cerebral ischemia.. Mol Med Rep.

[73] Hu Y, Li R, Yang H, Luo H, Chen Z (2015). Sirtuin 6 is essential for sodium sulfide-mediated cytoprotective effect in ischemia/reperfusion-stimulated brain endothelial cells.. J Stroke Cerebrovasc Dis.

[74] Ren L, Wei C, Li K, Lu Z (2018). LncRNA MALAT1up-regulates VEGF-A and ANGPT2 to promote angiogenesis in brain microvascular endothelial cells against oxygen-glucose deprivation via targeting miR-145.. Biosci Rep.

[75] Yang X, Zi XH (2018). LncRNA SNHG1 alleviates OGD induced injury in BMEC via miR-338/HIF-1alpha axis.. Brain Res.

[76] Ku JM, Taher M, Chin KY, Grace M, McIntyre P, Miller AA (2016). Characterisation of a mouse cerebral microvascular endothelial cell line (bEnd.3) after oxygen glucose deprivation and reoxygenation.. Clin Exp Pharmacol Physiol.

[77] Li WL, Yu SP, Chen D, Yu SS, Jiang YJ, Genetta T, Wei L (2013). The regulatory role of NF-kappaB in autophagy-like cell death after focal cerebral ischemia in mice.. Neuroscience.

[78] Friedrich V, Flores R, Sehba FA (2012). Cell death starts early after subarachnoid hemorrhage.. Neurosci Lett.

[79] Cui Y, Duan X, Li H, Dang B, Yin J, Wang Y, Gao A, Yu Z, Chen G (2016). Hydrogen Sulfide Ameliorates Early Brain Injury Following Subarachnoid Hemorrhage in Rats.. Mol Neurobiol.

[80] Shi W, Wei X, Wang Z, Han H, Fu Y, Liu J, Zhang Y, Guo J, Dong C, Zhou D, Zhou Q, Chen Y, Yi F (2016). HDAC9 exacerbates endothelial injury in cerebral ischaemia/reperfusion injury.. J Cell Mol Med.

[81] Tachibana M, Ago T, Wakisaka Y, Kuroda J, Shijo M, Yoshikawa Y, Komori M, Nishimura A, Makihara N, Nakamura K, Kitazono T (2017). Early Reperfusion After Brain Ischemia Has Beneficial Effects Beyond Rescuing Neurons.. Stroke.

[82] Ji J, Xiang P, Li T, Lan L, Xu X, Lu G, Ji H, Zhang Y, Li Y (2017). NOSH-NBP, a Novel Nitric Oxide and Hydrogen Sulfide-Releasing Hybrid, Attenuates Ischemic Stroke-Induced Neuroinflammatory Injury by Modulating Microglia Polarization.. Front Cell Neurosci.

[83] Li L, Liu F, Welser-Alves JV, McCullough LD, Milner R (2012). Upregulation of fibronectin and the alpha5beta1 and alphavbeta3 integrins on blood vessels within the cerebral ischemic penumbra.. Exp Neurol.

[84] Jiang MQ, Zhao YY, Cao W, Wei ZZ, Gu X, Wei L, Yu SP (2017). Long-term survival and regeneration of neuronal and vasculature cells inside the core region after ischemic stroke in adult mice.. Brain Pathol.

[85] Jais A, Solas M, Backes H, Chaurasia B, Kleinridders A, Theurich S, Mauer J, Steculorum SM, Hampel B, Goldau J, Alber J, Forster CY, Eming SA, Schwaninger M, Ferrara N, Karsenty G, Bruning JC (2016). Myeloid-Cell-Derived VEGF Maintains Brain Glucose Uptake and Limits Cognitive Impairment in Obesity.. Cell.

[86] Garbuzova-Davis S, Haller E, Williams SN, Haim ED, Tajiri N, Hernandez-Ontiveros DG, Frisina-Deyo A, Boffeli SM, Sanberg PR, Borlongan CV (2014). Compromised blood-brain barrier competence in remote brain areas in ischemic stroke rats at the chronic stage.. J Comp Neurol.

[87] Cai R, Pan C, Ghasemigharagoz A, Todorov MI, Forstera B, Zhao S, Bhatia HS, Parra-Damas A, Mrowka L, Theodorou D, Rempfler M, Xavier ALR, Kress BT, Benakis C, Steinke H, Liebscher S, Bechmann I, Liesz A, Menze B, Kerschensteiner M, Nedergaard M, Erturk A (2019). Panoptic imaging of transparent mice reveals whole-body neuronal projections and skull-meninges connections.. Nat Neurosci.

[88] Tam SJ, Richmond DL, Kaminker JS, Modrusan Z, Martin-McNulty B, Cao TC, Weimer RM, Carano RA, van Bruggen N, Watts RJ (2012). Death receptors DR6 and TROY regulate brain vascular development.. Dev Cell.

[89] McCabe C, Arroja MM, Reid E, Macrae IM (2018). Animal models of ischaemic stroke and characterisation of the ischaemic penumbra.. Neuropharmacology.

[90] Hou J, Wang S, Shang YC, Chong ZZ, Maiese K (2011). Erythropoietin employs cell longevity pathways of SIRT1 to foster endothelial vascular integrity during oxidant stress.. Curr Neurovasc Res.

[91] Liu Y, Jiang S, Yang PY, Zhang YF, Li TJ, Rui YC (2016). EF1A1/HSC70 Cooperatively Suppress Brain Endothelial Cell Apoptosis via Regulating JNK Activity.. CNS Neurosci Ther.

[92] Chen SL, Deng YY, Wang QS, Han YL, Jiang WQ, Fang M, Hu B, Wu ZX, Huang LQ, Zeng HK (2017). Hypertonic saline protects brain endothelial cells against hypoxia correlated to the levels of epidermal growth factor receptor and interleukin-1beta.. Medicine.

[93] Ge X, Li W, Huang S, Yin Z, Yang M, Han Z, Chen F, Wang H, Lei P, Zhang JN (2018). Increased miR-21-3p in injured brain microvascular endothelial cells following traumatic brain injury aggravates blood-brain barrier damage by promoting cellular apoptosis and inflammation through targeting MAT2B.. J Neurotrauma.

[94] Li W, Chen Z, Yan M, He P, Dai H (2016). The protective role of isorhamnetin on human brain microvascular endothelial cells from cytotoxicity induced by methylglyoxal and oxygen-glucose deprivation.. J Neurochem.

[95] Song J, Kang SM, Lee WT, Park KA, Lee KM, Lee JE (2014). The beneficial effect of melatonin in brain endothelial cells against oxygen-glucose deprivation followed by reperfusion-induced injury.. Oxid Med Cell Longev.

[96] Sun ZY, Wang FJ, Guo H, Chen L, Chai LJ, Li RL, Hu LM, Wang H, Wang SX (2019). Shuxuetong injection protects cerebral microvascular endothelial cells against oxygen-glucose deprivation reperfusion.. Neural Regen Res.

[97] Wu C, Zhao J, Chen Y, Li T, Zhu R, Zhu B, Zhang Y (2019). Tangeretin protects human brain microvascular endothelial cells against oxygen-glucose deprivation-induced injury.. J Cell Biochem.

[98] Zhang T, Wang H, Li Q, Fu J, Huang J, Zhao Y (2018). MALAT1 Activates the P53 Signaling Pathway by Regulating MDM2 to Promote Ischemic Stroke.. Cell Physiol Biochem.

[99] Zhang YM, Qu XY, Zhai JH, Tao LN, Gao H, Song YQ, Zhang SX (2018). Xingnaojing Injection Protects against Cerebral Ischemia Reperfusion Injury via PI3K/Akt-Mediated eNOS Phosphorylation.. Evid Based Complement Alternat Med.

[100] Yang G, Qian C, Wang N, Lin C, Wang Y, Wang G, Piao X (2017). Tetramethylpyrazine Protects Against Oxygen-Glucose Deprivation-Induced Brain Microvascular Endothelial Cells Injury via Rho/Rho-kinase Signaling Pathway.. Cell Mol Neurobiol.

[101] Shi S, Tang M, Li H, Ding H, Lu Y, Gao L, Wu Q, Zhou L, Fu Y, Xiao B, Zhang M (2018). X-box binding protein l splicing attenuates brain microvascular endothelial cell damage induced by oxygen-glucose deprivation through the activation of phosphoinositide 3-kinase/protein kinase B, extracellular signal-regulated kinases, and hypoxia-inducible factor-1alpha/vascular endothelial growth factor signaling pathways.. J Cell Physiol.

[102] Zhang L, Luo X, Chen F, Yuan W, Xiao X, Zhang X, Dong Y, Zhang Y, Liu Y (2018). LncRNA SNHG1 regulates cerebrovascular pathologies as a competing endogenous RNA through HIF-1alpha/VEGF signaling in ischemic stroke.. J Cell Biochem.

[103] Zhang X, Tang X, Liu K, Hamblin MH, Yin KJ (2017). Long Noncoding RNA Malat1 Regulates Cerebrovascular Pathologies in Ischemic Stroke.. J Neurosci.

[104] Yin KJ, Deng Z, Hamblin M, Xiang Y, Huang H, Zhang J, Jiang X, Wang Y, Chen YE (2010). Peroxisome proliferator-activated receptor delta regulation of miR-15a in ischemia-induced cerebral vascular endothelial injury.. J Neurosci.

[105] Abdullah Z, Rakkar K, Bath PM, Bayraktutan U (2015). Inhibition of TNF-alpha protects in vitro brain barrier from ischaemic damage.. Mol Cell Neurosci.

[106] Chen JL, Duan WJ, Luo S, Li S, Ma XH, Hou BN, Cheng SY, Fang SH, Wang Q, Huang SQ, Chen YB (2017). Ferulic acid attenuates brain microvascular endothelial cells damage caused by oxygen-glucose deprivation via punctate-mitochondria-dependent mitophagy.. Brain Res.

[107] Li H, Gao A, Feng D, Wang Y, Zhang L, Cui Y, Li B, Wang Z, Chen G (2014). Evaluation of the protective potential of brain microvascular endothelial cell autophagy on blood-brain barrier integrity during experimental cerebral ischemia-reperfusion injury.. Transl Stroke Res.

[108] Li Z, Li J, Tang N (2017). Long noncoding RNA Malat1 is a potent autophagy inducer protecting brain microvascular endothelial cells against oxygen-glucose deprivation/reoxygenation-induced injury by sponging miR-26b and upregulating ULK2 expression.. Neuroscience.

[109] Wang S, Han X, Mao Z, Xin Y, Maharjan S, Zhang B (2019). MALAT1 lncRNA Induces Autophagy and Protects Brain Microvascular Endothelial Cells Against Oxygen-Glucose Deprivation by Binding to miR-200c-3p and Upregulating SIRT1 Expression.. Neuroscience.

[110] Saito T, Nah J, Oka SI, Mukai R, Monden Y, Maejima Y, Ikeda Y, Sciarretta S, Liu T, Li H, Baljinnyam E, Fraidenraich D, Fritzky L, Zhai P, Ichinose S, Isobe M, Hsu CP, Kundu M, Sadoshima J (2019). An alternative mitophagy pathway mediated by Rab9 protects the heart against ischemia.. J Clin Invest.

[111] Engelhardt S, Huang SF, Patkar S, Gassmann M, Ogunshola OO (2015). Differential responses of blood-brain barrier associated cells to hypoxia and ischemia: a comparative study.. Fluids Barriers CNS.

[112] Kubli DA, Ycaza JE, Gustafsson AB (2007). Bnip3 mediates mitochondrial dysfunction and cell death through Bax and Bak.. Biochem J.

[113] Bellot G, Garcia-Medina R, Gounon P, Chiche J, Roux D, Pouyssegur J, Mazure NM (2009). Hypoxia-induced autophagy is mediated through hypoxia-inducible factor induction of BNIP3 and BNIP3L via their BH3 domains.. Mol Cell Biol.

[114] Wang HJ, Wei JY, Liu DX, Zhuang SF, Li Y, Liu H, Ban M, Fang WG, Cao L, Zhao WD, Chen YH (2018). Endothelial Atg7 Deficiency Ameliorates Acute Cerebral Injury Induced by Ischemia/Reperfusion.. Front Neurol.

[115] ElAli A, Hermann DM (2012). Liver X receptor activation enhances blood-brain barrier integrity in the ischemic brain and increases the abundance of ATP-binding cassette transporters ABCB1 and ABCC1 on brain capillary cells.. Brain Pathol.

[116] Han F, Chen YX, Lu YM, Huang JY, Zhang GS, Tao RR, Ji YL, Liao MH, Fukunaga K, Qin ZH (2011). Regulation of the ischemia-induced autophagy-lysosome processes by nitrosative stress in endothelial cells.. J Pineal Res.

[117] Wang YC, Zhou Y, Fang H, Lin S, Wang PF, Xiong RP, Chen J, Xiong XY, Lv FL, Liang QL, Yang QW (2014). Toll-like receptor 2/4 heterodimer mediates inflammatory injury in intracerebral hemorrhage.. Ann Neurol.

[118] Sukumari-Ramesh S, Laird MD, Singh N, Vender JR, Alleyne CH, Dhandapani KM (2010). Astrocyte-derived glutathione attenuates hemin-induced apoptosis in cerebral microvascular cells.. Glia.

[119] Dixon SJ, Lemberg KM, Lamprecht MR, Skouta R, Zaitsev EM, Gleason CE, Patel DN, Bauer AJ, Cantley AM, Yang WS, Morrison B, Stockwell BR (2012). Ferroptosis: an iron-dependent form of nonapoptotic cell death.. Cell.

[120] Li Q, Weiland A, Chen X, Lan X, Han X, Durham F, Liu X, Wan J, Ziai WC, Hanley DF, Wang J (2018). Ultrastructural Characteristics of Neuronal Death and White Matter Injury in Mouse Brain Tissues After Intracerebral Hemorrhage: Coexistence of Ferroptosis, Autophagy, and Necrosis.. Front Neurol.

[121] Liu M, Wilson NO, Hibbert JM, Stiles JK (2013). STAT3 regulates MMP3 in heme-induced endothelial cell apoptosis.. PLoS One.

[122] Kapitulnik J, Benaim C, Sasson S (2012). Endothelial Cells Derived from the Blood-Brain Barrier and Islets of Langerhans Differ in their Response to the Effects of Bilirubin on Oxidative Stress Under Hyperglycemic Conditions.. Front Pharmacol.

[123] Cardoso FL, Kittel A, Veszelka S, Palmela I, Toth A, Brites D, Deli MA, Brito MA (2012). Exposure to Lipopolysaccharide and/or Unconjugated Bilirubin Impair the Integrity and Function of Brain Microvascular Endothelial Cells.. PLoS ONE.

[124] Zhou XM, Zhang X, Zhang XS, Zhuang Z, Li W, Sun Q, Li T, Wang CX, Zhu L, Shi JX, Zhou ML (2014). SIRT1 inhibition by sirtinol aggravates brain edema after experimental subarachnoid hemorrhage.. J Neurosci Res.

[125] Fumoto T, Naraoka M, Katagai T, Li Y, Shimamura N, Ohkuma H (2019). The Role of Oxidative Stress in Microvascular Disturbances after Experimental Subarachnoid Hemorrhage.. Transl Stroke Res..

[126] Rodriguez C, Sobrino T, Agulla J, Bobo-Jimenez V, Ramos-Araque ME, Duarte JJ, Gomez-Sanchez JC, Bolanos JP, Castillo J, Almeida A (2017). Neovascularization and functional recovery after intracerebral hemorrhage is conditioned by the Tp53 Arg72Pro single-nucleotide polymorphism.. Cell Death Differ.

[127] Yan J, Li L, Khatibi NH, Yang L, Wang K, Zhang W, Martin RD, Han J, Zhang J, Zhou C (2011). Blood-brain barrier disruption following subarchnoid hemorrhage may be faciliated through PUMA induction of endothelial cell apoptosis from the endoplasmic reticulum.. Exp Neurol.

[128] Lyden PD, Lamb J, Kothari S, Toossi S, Boitano P, Rajput PS (2018). Differential effects of hypothermia on neurovascular unit determine protective or toxic results: Toward optimized therapeutic hypothermia.. J Cereb Blood Flow Metab.

[129] Li C, Bian Y, Feng Y, Tang F, Wang L, Hoi MPM, Ma D, Zhao C, Lee SMY (2019). Neuroprotective Effects of BHDPC, a Novel Neuroprotectant, on Experimental Stroke by Modulating Microglia Polarization.. ACS Chem Neurosci.

[130] Redzic ZB, Rabie T, Sutherland BA, Buchan AM (2015). Differential effects of paracrine factors on the survival of cells of the neurovascular unit during oxygen glucose deprivation.. Int J Stroke.

[131] Huang C, Dai C, Gong K, Zuo H, Chu H (2016). Apelin-13 protects neurovascular unit against ischemic injuries through the effects of vascular endothelial growth factor.. Neuropeptides.

[132] Zuo G, Zhang D, Mu R, Shen H, Li X, Wang Z, Li H, Chen G (2018). Resolvin D2 protects against cerebral ischemia/reperfusion injury in rats.. Mol Brain.

[133] El Amki M, Wegener S (2017). Improving Cerebral Blood Flow after Arterial Recanalization: A Novel Therapeutic Strategy in Stroke.. Int J Mol Sci.

[134] Kloner RA (2011). No-reflow phenomenon: maintaining vascular integrity.. J Cardiovasc Pharmacol Ther.

[135] Wolff J, Chao TI (2004). Cytoarchitectonics of non-neuronal cells in the central nervous system.. Adv Mol Cell Biol.

[136] Bartel H, Lametschwandtner A (2000). Regression of blood vessels in the ventral velum of Xenopus laevis Daudin during metamorphosis: light microscopic and transmission electron microscopic study.. J Anat.

[137] Chiu JJ, Chien S (2011). Effects of disturbed flow on vascular endothelium: pathophysiological basis and clinical perspectives.. Physiol Rev.

[138] Fogarty CE, Bergmann A, Steller H (2015). Chapter Nine - The Sound of Silence: Signaling by Apoptotic Cells.. Current Topics in Developmental Biology..

[139] Villringer A, Them A, Lindauer U, Einhaupl K, Dirnagl U (1994). Capillary perfusion of the rat brain cortex. An in vivo confocal microscopy study.. Circ Res.

[140] Kleinfeld D, Mitra PP, Helmchen F, Denk W (1998). Fluctuations and stimulus-induced changes in blood flow observed in individual capillaries in layers 2 through 4 of rat neocortex.. Proc Natl Acad Sci U S A.

[141] Erdener SE, Tang J, Sajjadi A, Kilic K, Kura S, Schaffer CB, Boas DA (2019). Spatio-temporal dynamics of cerebral capillary segments with stalling red blood cells.. J Cereb Blood Flow Metab.

[142] Lam CK, Yoo T, Hiner B, Liu Z, Grutzendler J (2010). Embolus extravasation is an alternative mechanism for cerebral microvascular recanalization.. Nature.

[143] Reeson P, Choi K, Brown CE (2018). VEGF signaling regulates the fate of obstructed capillaries in mouse cortex.. eLife.

[144] Franco CA, Jones ML, Bernabeu MO, Geudens I, Mathivet T, Rosa A, Lopes FM, Lima AP, Ragab A, Collins RT, Phng LK, Coveney PV, Gerhardt H (2015). Dynamic endothelial cell rearrangements drive developmental vessel regression.. PLoS Biol.

[145] Krueger M, Bechmann I, Immig K, Reichenbach A, Hartig W, Michalski D (2015). Blood-brain barrier breakdown involves four distinct stages of vascular damage in various models of experimental focal cerebral ischemia.. J Cereb Blood Flow Metab.

[146] Baldo C, Tanjoni I, Leon IR, Batista IF, Della-Casa MS, Clissa PB, Weinlich R, Lopes-Ferreira M, Lebrun I, Amarante-Mendes GP, Rodrigues VM, Perales J, Valente RH, Moura-da-Silva AM (2008). BnP1, a novel P-I metalloproteinase from Bothrops neuwiedi venom: biological effects benchmarking relatively to jararhagin, a P-III SVMP.. Toxicon.

[147] Kirby JE (2004). Anthrax lethal toxin induces human endothelial cell apoptosis.. Infect Immun.

[148] Nowatzki J, Sene RV, Paludo KS, Rizzo LE, Souza-Fonseca-Guimaraes F, Veiga SS, Nader HB, Franco CR, Trindade ES (2012). Brown spider (Loxosceles intermedia) venom triggers endothelial cells death by anoikis.. Toxicon.

[149] Lin JC, Lin SC, Chen WY, Yen YT, Lai CW, Tao MH, Lin YL, Miaw SC, Wu-Hsieh BA (2014). Dengue viral protease interaction with NF-kappaB inhibitor alpha/beta results in endothelial cell apoptosis and hemorrhage development.. J Immunol.

[150] Santoro MM, Samuel T, Mitchell T, Reed JC, Stainier DYR (2007). Birc2 (cIap1) regulates endothelial cell integrity and blood vessel homeostasis.. Nat Genet.

[151] Fisher M, Feuerstein G, Howells DW, Hurn PD, Kent TA, Savitz SI, Lo EH, Group S (2009). Update of the stroke therapy academic industry roundtable preclinical recommendations.. Stroke.

