# Supplementary material for: The impact of endothelial cell death in the brain and its role after stroke: A systematic review
Source: Cell Stress. 2019 Sep 25;3(11):330–47. doi: 10.15698/cst2019.11.203 (PMC6859425; doi:10.15698/cst2019.11.203)
Supplement: Supplementary file 1 [file ces-03-330-s01.pdf]

SUPPLEMENTAL DATA FOR

The impact of endothelial cell death in the brain and its role after stroke: A systematic review

Marietta Zille[1], Maulana Ikhsan[1], Yun Jiang[1,2], Josephine Lampe[1,2], Jan Wenzel[1,2], Markus Schwaninger[1,2]

Running Title: Brain endothelial cell death

[1]Institute for Experimental and Clinical Pharmacology and Toxicology, University of Lübeck, Lübeck, Germany

[2]DZHK (German Research Centre for Cardiovascular Research), partner site Hamburg/Lübeck/Kiel, Lübeck, Germany

Search Strategy

Database: Pubmed

Date: August 7, 2019

Strategy: #1 AND #2

#1 Brain endothelial cells [All Fields]

#2 cell death [All Fields] OR apoptosis [All Fields] OR necrosis [All Fields] OR necroptosis [All Fields] OR ferroptosis [All Fields] OR parthanatos [All Fields] OR pyroptosis [All Fields] OR autophagy [All Fields] OR NETosis [All Fields] OR anoikis [All Fields] OR autosis [All Fields] OR entosis

Supplemental Table 1. Suggested pathways of included stroke publications according to cell death subtype. 7-ADD: 7-aminoactinomycin D; AKT: protein kinase B; Atg7: autophagy related 7; BAK: BCL2 antagonist/killer 1; BAX: BCL2 associated X, apoptosis regulator; BBB: blood-brain barrier; BCL2: apoptosis regulator family; BCL-X_L: BCL2 like 1 (BCL2L1); BIM: BCL2-interacting mediator of cell death (also BCL2 like 11, BCL2L11); BMEC/BMVEC: brain microvascular EC; BNIP3: BCL2 interacting protein 3; CCK-8: Cell Counting Kit-8; CEC: cerebral endothelial cells; CHOP: C/EBP homologous protein; CMVEC: cerebral microvessel EC; DRP1: dynamin-related protein 1; EC: endothelial cells; EM: electron microscopy; GD: glucose deprivation; iPSC: induced pluripotent stem cells; ISL: in situ ligation; LC3: microtubule-associated protein 1A/1B-light chain 3; MALAT1: Metastasis-associated lung adenocarcinoma transcript 1; MCAO: middle cerebral artery occlusion; mTOR: mechanistic target of rapamycin; MTT: 3-(4,5-dimethylthiazol-2-yl)-2,5-diphenyltetrazolium bromide; OGD: oxygen-glucose deprivation; PARP: poly(ADP-ribose)-polymerase; phospho-JNK: phosphorylated c-Jun N-terminal kinase; phospho-p38: phosphorylated p38 mitogen-activated protein kinase; PI3K: phosphoinositide 3-kinase; PPAR: peroxisome proliferator-activated receptors; RBE4: rat brain endothelial cell line; RIP: receptor-interacting serine/threonine-protein kinase; TUNEL: TdT-mediated dUTP-biotin nick end labeling; VEGF: vascular endothelial growth factor; vWF: von Willebrand factor; WST: water-soluble tetrazolium; ZO-1: zona occludens.

Cell death subroutine	First Author	Year	PMID	Stroke subtype	Model	*in vitro/ in vivo*	Cell death measure	Pathway
apoptosis	Abdullah	2015	26546149	ischemic	human BMECs, transient OGD	*in vitro*	TUNEL, cleaved caspase-3/7 (Fig. 10, 11)	tumor necrosis factor inhibition
apoptosis	Ahmad	2019	31138990	ischemic	transient OGD in bEnd.3	*in vitro*	TUNEL (Fig. 2), cleaved caspase-3 (Fig. 2, 4)	complement C3a receptor, extracellular signal-regulated kinase (ERK), intracellular adhesion molecule-1 (ICAM-1)
apoptosis	Basuroy	2013	23576575	ischemic	glutamate toxicity in primary pig CMVEC	*in vitro*	cytoplasmic DNA fragments detected by ELISA (Fig. 2, 7, 10), floating cells (Fig. 3, 7), Ca^{2+} (Fig. 4), cytochrome c (Fig. 8)	n/a
apoptosis	Butt	2011	21356382	hemorrhagic	systemic application of cell-free hemoglobin in guinea pig	*in vivo*	cleaved caspase-3 (Fig. 8), rest on BBB	n/a
apoptosis	Cardoso	2012	22586454	hemorrhagic	unconjugated bilirubin in primary rat BMEC	*in vitro*	LDH, Hoechst, cleaved caspase-3 (Fig. 1), disruption of plasma membrane, invagination, apoptotic bodies (EM pics, Fig. 4)	n/a

Cell death subroutine	First Author	Year	PMID	Stroke subtype	Model	*in vitro/ in vivo*	Cell death measure	Pathway
apoptosis	Chen	2017	28072729	ischemic	transient OGD in bEnd.3	*in vitro*	MTT, Annexin A5-PE and 7-ADD, TUNEL (Fig. 1)	n/a
apoptosis	Chen	2016	27652091	ischemic	permanent OGD in primary rat BMVEC	*in vitro*	AnxA5/PI (Fig. 1)	n/a
apoptosis	Chen	2018	29327153	ischemic	transient OGD rat BMECs	*in vitro*	nuclear condensation with DAPI (Fig. 2), AnxA5/PI (Fig. 3), cleaved caspase-3, BAX, BCL2 (Fig. 4)	n/a
apoptosis	Cui	2016	26111628	hemorrhagic	rat autologous blood SAH	*in vivo*	vWF+TUNEL in ACA and MCA (Fig. 8), *in vitro:* only HUVEC	n/a
apoptosis	Cui	2019	30876978	ischemic	transient OGD in human BMEC	*in vitro*	MTT, LDH, caspase-3 (Fig. 1)	endothelial nitric oxide synthase (eNOS), PI3K/AKT,
apoptosis	ElAli	2012	21767321	ischemic	microvessels isolated from mouse transient MCAO	*in vivo*	Calpain-1/2 activity (Fig. 2), pJNK1/2 and cleaved caspase-3 (Fig. 5)	liver X receptor, ATP-binding cassette transporters (ABCG1)
apoptosis	Engelhar dt	2015	25879623	ischemic	RBE4, primary rat brain EC in OD and OGD	*in vitro*	MTT (Fig. 5), BNIP3 (Fig. 6), BAX, Beclin-1 (Fig. 7), LC3-II (Fig. 8)	n/a
apoptosis	Fang	2016	26887441	ischemic	OGD in BMEC	*in vitro*	AnxA5/PI (Fig. 2, 6), *in vivo*: only BBB dysruption	miRNA
apoptosis	Feng	2019	30828041	ischemic	transient OGD in bEnd.3	*in vitro*	MTT, Annexin A5/PI, cleaved caspase-3 (Fig. 1), in vivo: only in whole brain/neurons (Fig. 5-7)	VEGF, endoplasmic reticulum stress
apoptosis	Fu	2019	30962864	ischemic	transient OGD in human BMVEC	*in vitro*	CCK-8, Annexin A5/PI, EM to assess autophagy but no criteria mentioned (Fig. 1), LDH (Fig. 2), mTOR, Beclin-1, Bcl-2, Bax, LC3-II, cleaved caspase-3 (Fig. 4)	n/a
apoptosis	Friedrich	2013	24250830	hemorrhagic	SAH endovascular suture model in male and female rats	*in vivo*	TUNEL+Collagen IV-positive and cleaved caspase-3+Collagen IV-positive vascular cells (Fig. 5)	n/a

Cell death subroutine	First Author	Year	PMID	Stroke subtype	Model	*in vitro/ in vivo*	Cell death measure	Pathway
apoptosis	Friedrich	2012	22306092	hemorrhagic	SAH endovascular suture model in rats	*in vivo*	cleaved caspase-3+Collagen IV-positive vascular cells (Fig. 1), overall cell death with TUNEL, cleaved caspase-3 and fluorojade shown also	n/a
apoptosis	Fumoto	2019	30628008	hemorrhagic	microvascular endothelial cells after rat SAH endovascular perforation	*in vivo*	TUNEL+Collagen IV- or RECA-1-positive cells (Fig. 5)	n/a
apoptosis	Ge	2018	29695199	ischemic	permanent OGD in bEnd.3	*in vitro*	TUNEL+CD31-positive cells (Fig. 3), BCL2, cleaved caspase-3/9 (Fig. 3, 5)	miRNA, methionine adenosyltransferase 2B (MAT2B)
apoptosis	Han	2016	26231971	ischemic	rat transient MCAO	*in vivo*	swollen mitochondria, big vacuoles (EM pics, Fig 1), in vitro: MTT (Fig. 7, 12)	n/a
apoptosis	Hasegawa	2012	22183833	hemorrhagic	rat SAH endovascular perforation model	*in vivo*	TUNEL+vWF (Fig. 4), p-p38, p-JNK (Fig. 2) only in full extracts	n/a
apoptosis	He	2012	22944263	hemorrhagic	basilar artery of rat SAH endovascular perforation model	*in vivo*	TUNEL+vWF+ CHOP (Fig. 2), CHOP, BIM, BCL2, cleaved caspase-3 (Fig. 1) in whole basilar artery, TUNEL (Fig. 3) in whole basilar artery	C/EBP homologous protein (CHOP)
apoptosis	Hou	2011	21722091	ischemic	transient OGD in rat primary cerebral microvascular EC	*in vitro*	Trypan blue, TUNEL, Phosphatidylserine externalization (Fig. 1, 4), mitochondrial depolarization, cytochrome c release, BAD activation (Fig. 7), cleaved caspase-1/3 (Fig. 8)	sirtuin 1
apoptosis	Huang	2019	30518742	ischemic	transient OGD in primary rat BMEC	*in vitro*	CCK-8 (Fig. 1), Annexin A5/PI (Fig. 2)	n/a
apoptosis	Hunter	2019	31118073	hemorrhagic	thrombin in human BMEC from 21-year-old male and 26-year-old female who died from car accidents	*in vitro*	MTS, active caspase-3 (Fig. 1)	n/a

Cell death subroutine	First Author	Year	PMID	Stroke subtype	Model	*in vitro/ in vivo*	Cell death measure	Pathway
apoptosis	Imai	2017	27855593	ischemic	permanent OGD in human BMVEC	*in vitro*	WST-8/CCK-8 (Fig. 2), Hoechst 33342, TUNEL (Fig. 3), cleaved caspase-3/7 (Fig. 4), mitochondrial membrane potential (Fig. 5)	n/a
apoptosis	Imai	2019	30996325	hemorrhagic	Hemoglobin, ferrous ammonium sulfate, and hemin in human BMVEC	*in vitro*	PI (Fig. 1-3, 5), CCK-8, cleaved caspase-3 (Fig. 5), *in vivo*: only neuronal death assessed (Fig. 6)	n/a
apoptosis	Ji	2017	28603491	ischemic	microvessels from mouse transient MCAO, transient OGD in bEnd.3	both	*in vivo*: swollen mitochondria with vague crista and blurred membranes in ECs (EM pics, Fig. 4), *in vitro*: MTT and LDH (Fig. 8)	n/a
apoptosis	Jiang	2017	27514013	ischemic	mouse embolic permanent MCAO	*in vivo*	Glut-1^{+} cells, mostly not TUNEL^{+} (Fig. 5, 9, 10), EM: surviving microvessels and EC (Fig. 7) in infarct core	n/a
apoptosis	Kapitulnik	2012	22811666	hemorrhagic	bilirubin +/- high glucose in bEnd.3	*in vitro*	caspase activity (Fig. 2), cell counting (Tab. 1)	n/a
apoptosis	Kokubu	2017	28336435	ischemic	transient OGD in iPSC-derived BMECs	*in vitro*	Annexin A5 and 7-AAD (Fig. 4)	n/a
apoptosis	Ku	2016	27303049	ischemic	transient OGD in bEnd.3	*in vitro*	*in vitro*: MTT + cleaved caspase-3 (Fig. 5), *in vivo*: TUNEL and caspase-3 only in whole tissue (Fig. 2)	n/a
apoptosis	Ku	2016	27128638	ischemic	transient OGD in bEnd.3	*in vitro*	MTT (Fig. 5), cleaved caspase-3 (Fig. 6)	n/a
apoptosis	Lam	2010	20505729	ischemic	microvessels of microsphere embolism model in mice, young and aged animals	*in vivo*	cleaved caspase-3 (Fig. 4)	n/a
apoptosis	Li	2016	26578299	ischemic	permanent OGD +/- methylglyoxal in primary human BMEC	*in vitro*	MTT (Fig. 1), AnxA5/PI, TUNEL (Fig. 2), cleaved caspase-3 (Fig. 3)	n/a

Cell death subroutine	First Author	Year	PMID	Stroke subtype	Model	*in vitro/ in vivo*	Cell death measure	Pathway
apoptosis	Li	2013	23558089	ischemic	barrel cortex ischemic stroke in WT and p50 knockout mice	*in vivo*	Beclin-1+collagenIV-positive microvessels (Fig. 5), Beclin-1+ collagenIV+ TUNEL-positive microvessels (Fig. 6)	n/a
apoptosis	Li	2019	30839193	ischemic	transient OGD in bEnd.3	*in vitro*	MTT, LDH, caspase-3 (Fig. 9), in vivo: only in whole brain (Fig. 3)	n/a
apoptosis	Li	2019	30995438	ischemic	transient OGD in rat primary BMEC	*in vitro*	LDH, Annexin A5/PI (Fig. 5, 6)	n/a
apoptosis	Li	2019	31293373	ischemic	transient OGD in BMEC	*in vitro*	CCK-8 (Fig. 1, 3), Hoechst (Fig. 2), Annexin A5/PI, cleaved caspase-3, LC3-II (Fig. 2, 4), in vivo: only in whole tissue (Fig. 6)	SNHG12, PI3K/AKT/mTOR
apoptosis	Liao	2016	27885275	ischemic	transient OGD in bEnd.3, rat transient MCAO	both	*in vitro*: MTT, LDH (Fig. 1), Hoechst, Acridin Orange+ ethidium bromide (Fig. 2), cleaved caspases and BCL2 only in HUVEC, *in vivo*: TUNEL+CD31 (Fig. 6)	n/a
apoptosis	Liu	2013	23967200	hemorrhagic	hemin in Human brain vascular endothelial cells (HBVEC)	*in vitro*	MTT, TUNEL (Fig. 6)	signal transducer and activator of transcription 3
apoptosis	Liu	2016	27324700	ischemic	permanent OGD in bEnd.3 cells	*in vitro*	AnxA5/PI, cleaved caspase-3/9 (Fig. 3), pJNK, c-JUN (Fig. 4), SP600125 - JNK inhibitor (Fig. 5)	elongation factor 1-alpha 1, heat shock 70 kDa protein 8, JNK
apoptosis	Liu	2017	28367097	ischemic	rat permanent MCAO by electrocoagulation, permanent OGD in primary brain vascular endothelial cells (pBVEC) +/- 48h of low oxygen and nutrition (LON)	both	*in vivo*: VIII+TUNEL (Fig. 3), *in vitro*: MTT (Fig. 4/5), cytochrome c, cleaved caspase-3, BAX, BAK, BCL2 only in OGD+LON (Fig. 6-8), TUNEL, PI (Fig. 7, 8)	hypoxia-inducible factor 1α

Cell death subroutine	First Author	Year	PMID	Stroke subtype	Model	*in vitro/ in vivo*	Cell death measure	Pathway
apoptosis	Lok	2011	21534958	ischemic	transient OGD in human BMEC	*in vitro*	mitochondrial membrane potential, MTT (Fig. 4)	n/a
apoptosis	Luo	2017	28252551	ischemic	transient OGD in bEnd.3	*in vitro*	MTT, LDH (Fig. 2), Hoechst/PI (Fig. 3), AnxA5/PI, cleaved caspase-3, RIPK1, RIPK3, Necrostatin-1 (Fig. 4)	necroptosis mechanism
apoptosis	Luo	2019	30911000	ischemic	transient OGD in human BMEC	*in vitro*	MTT, Annexin A5/PI (Fig. 3), in vivo: no EC death assessed	PI3K, AKT
apoptosis	Lv	2016	26306919	ischemic	transient OGD in murine brain endothelial cells (mBEC)	*in vitro*	WST (like MTT, Fig. 1, 4), LDH, AnxA5/PI (Fig. 1), intracellular Ca^{2+} increase (Fig. 2)	n/a
apoptosis	Mey	2013	23816753	ischemic	rat transient and permanent MCAO, brain microvascular line hCMEC/D3	*in vivo*	ISL assay for DNA fragmentation (Fig. 4), cleaved caspase-3 (Fig. 5), *in vitro*: only VEGF, no stroke	VEGF increases PARP1 expression → DNA repair
apoptosis	Palmela	2011	21463246	hemorrhagic	uncojugated bilirubin in human BMEC and primary brain EC	*in vitro*	cleaved caspase-3, Hoechst, LDH (Fig. 1)	n/a
apoptosis	Palmela	2015	25821432	hemorrhagic	uncojugated bilirubin in human BMEC	*in vitro*	Hoechst (Fig. 1), caspase-3 activity (Fig. 2), decrease in mitochondrial cristae and ribosomes, detached cellular fragments (EM pics, Fig. 3)	n/a
apoptosis	Pang	2017	27796945	hemorrhagic	SAH in mice by endovascular perforation	*in vivo*	TUNEL + Lectin (Fig. 6), BAX, BCL2, cleaved caspase-3 from complete tissue	apolipoprotein E
apoptosis	Pang	2016	27463015	hemorrhagic	SAH in mice by endovascular perforation	*in vivo*	TUNEL + Lectin (Fig. 7), BAX, BCL2, cleaved caspase-3 from complete tissue	n/a
apoptosis	Pang	2018	30622670	ischemic	transient OGD in human BMVEC	*in vitro*	CCK-8 (Fig. 1), Annexin A5/PI, BCL2, cleaved caspase-3, autophagosome (EM), mTOR, Beclin-1 (Fig. 3), *in vivo*: no cell death assessed	n/a
apoptosis	Qi	2016	27388935	ischemic	microvessels from rat transient MCAO	*in vivo*	TUNEL (Fig. 6), *in vitro*: only BBB permeability	n/a

Cell death subroutine	First Author	Year	PMID	Stroke subtype	Model	*in vitro/ in vivo*	Cell death measure	Pathway
apoptosis	Rakkar	2016	26527181	ischemic	transient OGD in human BMEC	*in vitro*	intracellular calcium, cleaved caspase-3/7 (Fig. 1, 6), TUNEL (Fig. 2, 8)	nicotinamide adenine dinucleotide phosphate (NADPH) oxidase, protein kinase C-alpha, urokinase plasminogen activator
apoptosis	Ran	2011	21960347	ischemic	transient OGD in primary rat CMVEC	*in vitro*	MTT, LDH (Fig. 4), cleaved caspase-3, BCL2/BAX (Fig. 7), in vivo: only in whole tissue	n/a
apoptosis	Rodrigue z	2017	27768124	ischemic/ hemorrhagic	Mouse ICH collagenase model in Arg72Pro p53 KI mice, 1, 3 or 6h of OGD in prim. brain endothelial cells from Arg72Pro p53 KI mice	both	in vivo: in perihematoma TUNEL+CD31 at 24h (Fig. 3), in vitro: AnxA5 (Fig. 3)	n/a
apoptosis	Ruan	2019	30607811	ischemic	permanent OGD in primary rat BMVEC	*in vitro*	LDH, TUNEL (Fig. 2, 5), *in vivo*: only BBB dysfunction	MALAT1/ cAMP response element binding/ peroxisome proliferator-activated receptor γ co-activator 1α/PPARγ
apoptosis	Shi	2018	30317635	ischemic	transient OGD in primary rat BMEC	*in vitro*	MTS assay (Fig. 3), Annexin A5/PI (Fig. 4), cleaved caspase-3 (Fig. 7)	X-box binding protein l, hypoxia-inducible factor 1α, VEGF, phosphoinositide 3-kinase,
apoptosis	Song	2014	25126203	ischemic	transient OGD in bEnd.3	*in vitro*	MTT, LDH, PI (Fig. 1), pJNK, BAX (Fig. 5)	n/a
apoptosis	Sukumar i-Ramesh	2010	20737478	hemorrhagic	hemin in bEnd.3 and in primary human BMVEC	*in vitro*	MTT (Fig. 1, 2, 4, 5), deferoxamine, trolox (Fig. 1), LDH, AnxA5 (Fig. 2), cleaved caspase-3 (Fig. 3, 4), z-VAD treatment (Fig. 3)	n/a

Cell death subroutine	First Author	Year	PMID	Stroke subtype	Model	*in vitro/ in vivo*	Cell death measure	Pathway
apoptosis	Sun	2019	30688264	ischemic	transient OGD in bEnd.3	*in vitro*	CCK-8, LDH, cleaved caspase-3, BCL-2 (Fig. 1)	n/a
apoptosis	Tian	2013	23344049	ischemic	transient OGD in primary rat BMEC	*in vitro*	WST-8 (MTT, Fig. 2), Hoechst nuclear staining (Fig. 4), Annexin A5/7-AAD (Fig. 5), BCL-2 (Fig. 6, 7)	n/a
apoptosis	Tian	2013	23948104	ischemic	transient OGD in primary rat BMEC	*in vitro*	Annexin A5/7-AAD (Fig. 4), Hoechst33258/PI (Fig. 5), *in vivo*: no cell death assessed	n/a
apoptosis	Tian	2018	28987818	ischemic	transient OGD in primary rat BMEC	*in vitro*	WST-8 (MTT, Fig. 1), Annexin A5/7-AAD (Fig. 2), BCL-2 (Fig. 4)	n/a
apoptosis	Tu	2016	26111627	ischemic	transient OGD. in human brain microvascular endothelial cell line (HMEC)	*in vitro*	XTT (MTT), cleaved caspase-3 (Fig. 6, 7), *in vivo*: only in whole tissue	AKT
apoptosis	Wang	2018	29424909	ischemic	rat permanent MCAO, permanent OGD in cerebrovascular EC (VEC)	both	*in vivo*: Annexin A5/PI in vascular intima (Fig. 2), BCL-2 (Fig. 3), *in vitro*: AnnexinA5/PI, BCL-2 (Fig. 4)	n/a
apoptosis	Wu	2014	24352801	ischemic	transient OGD in CEC	*in vitro*	CCK-8 (MTT), LDH, mitochondrial membrane potential, cleaved caspase-3, (Fig. 1-3, 10)	PPARγ
apoptosis	Wu	2019	30260010	ischemic	transient OGD in human BMEC	*in vitro*	MTT (Fig. 1, 5), Annexin A5/PI (Fig. 4), cleaved caspase-3 (Fig. 4, 5), pJNK, SP600125 - JNK inhibitor (Fig. 5)	n/a
apoptosis	Xiang	2017	28844675	ischemic	transient OGD in human BMEC	*in vitro*	MTT, LDH (Fig. 2, 4), TUNEL (Fig. 2)	let-7i, TLR4
apoptosis	Xu	2017	29039513	ischemic	permanent OGD in bEnd.3	*in vitro*	CCK-8 (MTT), Annexin A5/PI (Fig. 1, 4)	PPARγ, Birc5
apoptosis	Xu	2019	30859754	hemorrhagic	hemin in bEnd.3	*in vitro*	LDH, Annexin A5/PI (Fig. 4, 6)	nucleotide-binding oligomerization domain-like receptor

Cell death subroutine	First Author	Year	PMID	Stroke subtype	Model	*in vitro/ in vivo*	Cell death measure	Pathway
								with a pyrin domain 3 (NLRP3) inflammasome
apoptosis	Yan	2011	21586287	hemorrhagic	rat SAH endovascular perforation model	*in vivo*	BAX, BAK, DRP1, TUNEL (Fig. 2), DRP1 western blot in whole hippocampus	PUMA
apoptosis	Yang	2017	27380043	ischemic	permanent OGD in primary rat BMEC	*in vitro*	shrunken cell body, condensed cytoplasm, damaged mitochondria (EM pics, Fig. 3), MTT, LDH (Fig. 4), acridine orange/ethidium bromide (AO/EB) (Fig. 5), Annexin A5/PI (Fig. 6), cleaved caspase-3 (Fig. 9)	Rho/ROCK
apoptosis	Yang	2018	29203245	ischemic	permanent OGD in bEnd.3	*in vitro*	cleaved caspase-3, CCK-8 (MTT) (Fig. 2), BIM, BAX (Fig. 3), *in vivo*: no cell death measured	KLF4
apoptosis	Yang	2012	25722681	ischemic	transient OGD in rat BMEC	*in vitro*	MTT (Fig. 1), Annexin A5/PI (Fig. 3), BCL-2, cleaved caspase-3 (Fig. 4)	HIF1α
apoptosis	Yang	2018	30414401	ischemic	permanent OGD in primary mouse BMEC	*in vitro*	MTT (Fig. 1, 2, 4), cleaved caspase-3 (Fig. 2, 4), BCL2, BAX (Fig. 2, 5)	SNHG1, miR-338, HIF-1α
apoptosis	Yin	2010	20445066	ischemic	transient OGD in mouse CEC	*in vitro*	LDH, Trypan Blue (Fig. 1, 3), Golgi fragmentation GRASP65 (Fig. 2), cleaved caspase-3 (Fig. 3), BCL-2 (Fig. 4, 6), BCL-X_L (Fig. 6), *in vivo*: cell death only in whole tissue	miR-15a, PPARδ
apoptosis	Yu	2017	29311781	ischemic	rat permanent MCAO by electrocoagulation, permanent OGD in rat BMVEC	both	*in vivo*: impaired microvscular structure, BBB (H&E, EM pics, Fig. 2), Beclin, LC3-II in whole tissue only, *in vitro*: CCK-8 and Annexin A5/PI (Fig. 5)	n/a

Cell death subroutine	First Author	Year	PMID	Stroke subtype	Model	*in vitro/ in vivo*	Cell death measure	Pathway
apoptosis	Yu	2019	31105826	ischemic	transient OGD in rat BMEC	*in vitro*	MTT (Fig. 2), Hoechst, Annexin A5/PI (Fig. 5), Bcl-2, Bax, caspase-3 (Fig. 6)	n/a
apoptosis	Zhang	2017	28347817	ischemic	permanent OGD in rat BMEC	*in vitro*	Hoechst, MTT (Fig. 1), TUNEL, BCL-2, BAX (Fig. 3)	WNT/β-catenin
apoptosis	Zhang	2018	29377234	ischemic	permanent OGD in primary mouse BMEC	*in vitro*	MTT, cleaved caspase-3 (Fig. 3, 4, 6, 7), Annexin A5/PI (Fig. 3), *in vivo*: no cell death assessed	SNHG1, HIF1α
apoptosis	Zhang	2017	28093478	ischemic	permanent OGD in primary mouse BMEC	*in vitro*	MTT, LDH, cleaved caspase-3 (Fig. 2), BIM, BAX (Fig. 5), *in vivo*: only in whole tissue	Malat1
apoptosis	Zhang	2016	27630541	ischemic	permanent OGD in bend.3	*in vitro*	LDH (Fig. 3, 6), TUNEL (Fig. 3), cleaved caspase-3, BAX, BCL-2 (Fig. 4, 6)	ENOPH1
apoptosis	Zhang	2018	30419554	ischemic	transient OGD in human HBMEC	*in vitro*	BAX (Fig. 3, 4), Annexin A5/PI (Fig. 4), *in vivo*: only whole tissue	MALAT1/MDM2/p53
apoptosis	Zhang	2019	30787267	ischemic	transient and permanent OGD in rat BMEC	*in vitro*	MTT (Fig. 1), LDH (Fig. 2), 3-MA (Fig. 2, 3), Annexin A5/PI (Fig. 3), AO/MDC (acridine orange/ monodansycadaverine, autophagosomes, Fig. 4)	n/a
apoptosis	Zhang	2018	30158991	ischemic	transient OGD in human BMEC	*in vitro*	MTT, Annexin A5/PI, DAPI (Fig. 7), cleaved caspase-3, mitochondrial membrane potential (Fig. 8), *in vivo*: only in whole brain	n/a
apoptosis	Zhou	2014	24482345	hemorrhagic	cisternal injection of autologous blood model of SAH in rat	*in vivo*	TUNEL in microvasculature (Fig. 5, Tab. 1), other in whole cortex	n/a
apoptosis	Zhou	2019	30408478	ischemic	permanent OGD in BMEC	*in vitro*	MTT (Fig. 1, 2, 4), cleaved caspase-3 (Fig. 2, 4), BCL-X_L, BAX (Fig. 3, 6)	NEAT1 , miR-377, SIRT1
apoptosis	Zuo	2018	29439730	ischemic	rat transient MCAO	*in vivo*	TUNEL+vWF (Fig. 3)	n/a

Cell death subroutine	First Author	Year	PMID	Stroke subtype	Model	*in vitro/ in vivo*	Cell death measure	Pathway
autophagy	Bake	2019	30287160	ischemic	human BMEC transient OGD, microvessels of transient MCAO in female rats	*in vitro*	LDH, Calcein, Rapamycin treatment (Fig. 1), *in vivo*: only vessel length and diameter assessed	n/a
autophagy (mitophagy), apoptosis excluded!	Chen	2017	28438530	ischemic	transient OGD in BMEC	*in vitro*	MTT, BAX, BCL2, cleaved caspase-3 (Fig. 1), mitochondrial fission/DRP1 (Fig. 2), Rapamycin, chloroquine, 3-methyladenine treatment + MTT, LC3-II (Fig. 3), Mdivi-1 treatment + MTT, DRP1, LC3-II (Fig. 4)	reactive oxygen species, DRP1, autophagy
autophagy	Engelhardt	2015	25879623	ischemic	RBE4, primary rat brain EC in OD and OGD	*in vitro*	MTT (Fig. 5), BNIP3 (Fig. 6), BAX, Beclin-1 (Fig. 7), LC3-II (Fig. 8)	n/a
autophagy	Fu	2019	30962864	ischemic	transient OGD in human BMVEC	*in vitro*	CCK-8, Annexin A5/PI, EM to assess autophagy but no criteria mentioned (Fig. 1), LDH (Fig. 2), mTOR, Beclin-1, Bcl-2, Bax, LC3-II, cleaved caspase-3 (Fig. 4)	n/a
autophagy	Garbuzova-Davis	2014	24610730	ischemic	rat transient MCAO	*in vivo*	EM examination of microvasculature, formation of autophagosomes, large vacuoles (Fig. 1, 2), Beclin-1 (Fig. 4, 5)	n/a
autophagy	Han	2011	21392095	ischemic	rat microvessels from permanent microsphere embolism model	*in vivo*	in microvessels: Cathepsin B + vWF (Fig. 5), Cathepsin B +ZO-1 (Fig. 7), LC3-II (Fig 5, 6) not EC-specific, *in vitro*: OGD in EA.hy 926 cell line	n/a
autophagy	Li	2014	25070048	ischemic	transient OGD in BMVEC	*in vitro*	rapamycin, lithium carbonate, 3-methyladenine treatment and LC3-II (Fig. 1), Hoechst (Fig. 2), *in vivo*: no cell death in EC investigated	n/a

Cell death subroutine	First Author	Year	PMID	Stroke subtype	Model	*in vitro/ in vivo*	Cell death measure	Pathway
autophagy	Li	2013	23558089	ischemic	barrel cortex ischemic stroke in WT and p50 knockout mice	*in vivo*	Beclin-1+collagenIV-positive microvessels (Fig. 5), Beclin-1+ collagenIV+ TUNEL-positive microvessels (Fig. 6)	n/a
autophagy	Li	2017	28433650	ischemic	transient OGD in mouse primary BMEC	*in vitro*	LC3-II, Trypan Blue, PI (Fig. 1, 4), 3-methyladenine treatment, lithium carbonate (Fig. 1)	Malat1, miRNA, Unc-51 like autophagy activating kinase 2
autophagy	Lonati	2019	31370282	ischemic	transient OGD in RBE4	*in vitro*	Beclin-1 (Fig. 6), LC3-II (Fig. 6, 7)	Lipid peroxidation, lipophagy
autophagy	Palmela	2012	22590454	hemorrhagic	uncojugated bilirubin in human BMEC	*in vitro*	LC3-II (Fig. 7)	n/a
autophagy	Pang	2018	30622670	ischemic	transient OGD in human BMVEC	*in vitro*	CCK-8 (Fig. 1), Annexin A5/PI, BCL2, cleaved caspase-3, autophagosome (EM), mTOR, Beclin-1 (Fig. 2), *in vivo*: no cell death assessed	n/a
autophagy (mitophagy)	Qi	2019	31152817	ischemic	transient OGD in bEnd.3	*in vitro*	Drp1 (Fig. 4)	n/a
autophagy	Shi	2016	26865248	ischemic	rat transient MCAO, transient OGD in BMVEC	both	*in vivo*: endothelial cells and their nucleus were swollen and deformed (EM pics, Fig. 3), *in vitro*: flow cytometry - marker not mentioned (Fig. 4), LC3-II, rapamycin treatment (Fig. 6)	histone deacetylase 9
autophagy	Wang	2018	30555402	ischemic	mouse transient MCAO in endothelial-specific Atg7 knockout (Atg7 eKO) vs WT, transient OGD in human BMEC	both	*in vivo*: endothelial Atg7 KO (Fig. 1), CD31 (Fig. S1), *in vitro*: CCK-8, shAtg7 (Fig. S3)	Atg7, NF-kB
autophagy	Wang	2019	30496821	ischemic	permanent OGD in bEnd.3	*in vitro*	LC3 (Fig. 1, 2, 4, 6), MTT, LDH (Fig. 2, 4, 6)	MALAT1, miR-200c-3p, SIRT1

Cell death subroutine	First Author	Year	PMID	Stroke subtype	Model	*in vitro/ in vivo*	Cell death measure	Pathway
autophagy	Yan	2011	21586287	hemorrhagic	rat SAH endovascular perforation model	*in vivo*	BAX, BAK, DRP1, TUNEL (Fig. 2), DRP1 western blot in whole hippocampus	PUMA
autophagy	Zhang	2018	29524637	ischemic	transient OGD in rat BMEC	*in vitro*	LDH, MTT (Fig. 2), LC3-II (Fig. 6A), *in vivo*: cell death only in whole tissue	n/a
autophagy	Zhang	2019	30787267	ischemic	transient and permanent OGD in rat BMEC	*in vitro*	MTT (Fig. 1), LDH (Fig. 2), 3-MA (Fig. 2, 3), Annexin A5/PI (Fig. 3), AO/MDC (acridine orange/ monodansycadaverine, autophagosomes, Fig. 4)	n/a
lysosome-dependent cell death	ElAli	2012	21767321	ischemic	microvessels isolated from mouse transient MCAO	*in vivo*	Calpain-1/2 activity (Fig. 2), pJNK1/2 and cleaved caspase-3 (Fig. 5)	liver X receptor, ATP-binding cassette transporters (ABCG1)
lysosome-dependent cell death	Han	2011	21392095	ischemic	rat microvessels from permanent microsphere embolism model	*in vivo*	in microvessels: Cathepsin B + vWF (Fig. 5), Cathepsin B +ZO-1 (Fig. 7), LC3-II (Fig 5, 6) not EC-specific, *in vitro*: OGD in EA.hy 926 cell line	n/a
necroptosis	Abdul	2018	29909454	ischemic/ hemorrhagic	rat primary BMVEC of diabetic rats, transient OGD + iron	*in vitro*	MTT (Fig. 5A), RIP3 (Fig. 6), *in vivo*: no cell death assessed	n/a
necroptosis	Luo	2017	28252551	ischemic	transient OGD in bEnd.3	*in vitro*	MTT, LDH (Fig. 2), Hoechst/PI (Fig. 3), AnxA5/PI, cleaved caspase-3, RIPK1, RIPK3, Necrostatin-1 (Fig. 4)	necroptosis mechanism
unspecific	Cao	2016	26915982	ischemic	permanent OGD in bEnd.3	*in vitro*	MTT (Fig. 2)	n/a
unspecific	Ceruti	2011	21672581	ischemic	permanent GD or OGD in rat brain capillary EC	*in vitro*	LDH, PI (Fig. 3)	n/a
unspecific	Chen	2018	29963617	ischemic	mouse embolic model of focal cerebral ischemia	*in vivo*	endothelial density (CD31+, Fig. 5) caused by laminin degradation	laminin degradation

Cell death subroutine	First Author	Year	PMID	Stroke subtype	Model	*in vitro/ in vivo*	Cell death measure	Pathway
unspecific	Cheng	2019	30414726	ischemic	rat brain EC transient OGD, rat transient MCAO	*in vitro*	phase-contrast (morphology), CCK-8 (Fig. 4), only whole brain neuronal death, microvessels transcription factor expression, *in vivo*: only BBB	n/a
unspecific	Chu	2017	28402976	ischemic	mouse transient MCAO	*in vivo*	swollen capillary EC (EM pictures, Fig. 7), in cultured astrocytes: no increase in p-JNK and p-p38 (Fig. 8)	n/a
unspecific	Clark	2012	23082218	ischemic	permanent OGD in primary rat brain EC	*in vitro*	tetrazolium-based CellTiter 96 Aqueous One Solution assay (Fig. 5), *in vivo*: only BBB	n/a
unspecific	Dong	2018	30120860	hemorrhagic	primary mouse brain vascular EC from AngII- + L-NAME-induced hypertension and HBMECs treated with thrombin	*in vitro*	Annexin A5 (flow cytometry) (Fig. 2, 4)	siFENDRR, miR-126, VEGFA
unspecific	Fu	2014	24930357	ischemic	OGD in bEnd.3	*in vitro*	LDH (Fig. 6), *in vivo*: only BBB	n/a
unspecific	Gao	2010	20496198	ischemic	rat permanent MCAO	*in vivo*	Enlargement of the EC nucleus and cytoplasm, tight junction opening and swelling mitochondria in MCAO animals (EM pictures, Fig. 2)	n/a
unspecific	Guo	2010	19861973	ischemic	transient OGD in bEnd.3	*in vitro*	MTT (Fig. 1), LDH (Fig. 2)	n/a
unspecific	Guo	2010	20664263	ischemic	Transient OGD in SV40 transformed human BMEC	*in vitro*	MTT and LDH (Fig. 3)	n/a
unspecific	Hawkins	2015	25669912	hemorrhagic	thrombin in mouse primary BMEC	*in vitro*	PI (Fig. 1) no effect of thrombin on cellular demise	n/a
unspecific	Hu	2015	25543188	ischemic	transient OGD in bEnd.3	*in vitro*	MTT (Fig. 1), LDH (Fig. 1, 4)	sirtuin 6
unspecific	Huang	2016	27592408	ischemic	transient OGD in prim. rat cortical EC	in vitro	*in vitro*: MTT (Fig. 6), *in vivo*: increase in CD31 staining (Fig. 4, 5)	n/a
unspecific	Hwang	2019	31024439	ischemic	rat transient MCAO, transient OGD in bEnd.3	both	*in vivo*: CD31 (Fig. 5), *in vitro:* Trypan blue, WST-8 (Fig. 7)	n/a

Cell death subroutine	First Author	Year	PMID	Stroke subtype	Model	*in vitro/ in vivo*	Cell death measure	Pathway
unspecific	Iwata	2018	30092231	hemorrhagic	hemoglobin or collagenase in human BMVEC	*in vitro*	PI (Fig. 2, 3)	n/a
unspecific	Ji	2013	24028618	ischemic	permanent OGD in rat BMEC	*in vitro*	MTT (Table 1)	n/a
unspecific	Jiang	2017	29146880	ischemic	mouse transient MCAO	*in vivo*	CD31/microvessel density in peri-infarct (Fig. 3) but without control, TUNEL only in whole tissue	n/a
unspecific	Jung	2010	20560878	ischemic	transient OGD in bEnd.3	*in vitro*	LDH (Fig. 1)	n/a
unspecific	Krupinski	2012	23227823	ischemic	rat transient MCAO	*in vivo*	$CD31^{+}$ microvessels, no comparison to sham (Fig. 5)	n/a
unspecific	Kuntz	2014	24333620	ischemic	transient OGD in primary bovine brain capillary EC	*in vitro*	PI (Fig. 2), LDH only mentioned in the text, not shown (p.50)	n/a
unspecific	Lecht	2010	20012228	ischemic	transient OGD in bEnd.3	*in vitro*	LDH (Fig. 5)	extracellular signal–regulated kinase (ERK) phosphorylation
unspecific	Lee	2018	29870781	ischemic	glutamate toxicity on conditionally immortalized rat brain capillary EC cell line (TR-BBB cells)	*in vitro*	MTT (Fig. 1, 2)	n/a
unspecific	Li	2012	22056225	ischemic	mouse transient MCAO	*in vivo*	decreased CD31 in core, increased in penumbra (Fig. 2), rest on angiogenesis	n/a
unspecific	Li	2014	25618978	ischemic	permanent OGD in rat primary BMEC or OGD in astrocytes and treatment of BMEC with conditioned media from astrocytes and vice versa	*in vitro*	MTT (Fig. 1)	n/a
unspecific	Li	2012	22472112	ischemic	transient OGD in BMEC, and conditioned medium from BMEC on hippocampal neurons	*in vitro*	phase contrast (Fig. 2), rest in neurons	n/a
unspecific	Li	2019	31290452	ischemic	transient OGD in in primary rat BMEC	*in vitro*	CCK-8 (Fig. 3)	n/a

Cell death subroutine	First Author	Year	PMID	Stroke subtype	Model	*in vitro/ in vivo*	Cell death measure	Pathway
unspecific	Liu	2012	23071504	ischemic	OGD in bEnd.3	*in vitro*	LDH (Fig. 7)	Nicotinamide phosphoribosyltransferase
unspecific	Long	2018	30233065	ischemic	transient OGD in brain microvascular EC	*in vitro*	MTT, Trypan blue (Fig. 1, 5)	SNHG12, miR199a, VEGF
unspecific	Lyden	2018	30461327	ischemic	transient OGD in primary rat brain EC	*in vitro*	MTT, LDH (Fig. 2, 4)	n/a
unspecific	Mao	2018	29533123	ischemic	permanent OGD in primary mouse BMEC	*in vitro*	live/dead staining, LDH (Fig. 6), *in vivo:* no cell death investigated	n/a
unspecific	Orsini	2018	30354247	ischemic	transient OGD in human BMEC	*in vitro*	PI (Fig. 5), *in vivo*: no cell death assessed	platelet activation, mannose-binding lectin interaction with interleukin-1
unspecific	Page	2016	27724968	ischemic	chemical hypoxia with cobalt chloride, permanent OD and OGD in brain microvascular line hCMEC/D3 and IMR90 stem-cell derived human brain microvascular endothelial cell lines	*in vitro*	Cell density by Trypan blue, MTT (Fig. 1, 3, 4)	n/a
unspecific	Panickar	2015	24773045	ischemic	permanent OGD in bEnd.3	*in vitro*	cell swelling (Fig. 1, 2)	n/a
unspecific	Park	2013	23374901	ischemic	transient OGD in bEnd.3	*in vitro*	LDH (Fig. 1-3)	methallothionein, signal transducer and activator of transcription 3
unspecific	Redzic	2015	24206924	ischemic	permanent OGD in primary rat brain EC	*in vitro*	LDH (Fig. 3-6)	n/a
unspecific	Ren	2018	30038058	ischemic	permanent OGD in primary mouse BMEC	*in vitro*	MTT (Fig. 1)	LncRNA-MALAT, miR-145, VEGF, angiopoietin-2
unspecific	Salvador	2015	26347611	ischemic	transient OGD in mouse cerebrovascular EC (cEND)	*in vitro*	OGD: LDH (Fig. 3), intracellular Ca^{2+} (Fig. 11)	n/a

Cell death subroutine	First Author	Year	PMID	Stroke subtype	Model	*in vitro/ in vivo*	Cell death measure	Pathway
unspecific	Shin	2018	29574357	ischemic	transient OGD in bEnd.3	*in vitro*	LDH, MTT (Fig. 4,5)	adenosine triphosphate-binding cassette transporter (ABCG2)
unspecific	Song	2010	20125184	ischemic	transient OGD in bEnd.3	*in vitro*	Calcein AM/ethidium homodimer, LDH (Fig. 6), *in vivo*: no cell death assessed	n/a
unspecific	Sun	2019	30422362	ischemic	transient OGD in human brain microvascular endothelial cells (HBEC-5i)	*in vitro*	CCK-8, MTT (Fig. 1, 2, 4), *in vivo*: only BBB	calcium/calmodulin-dependent protein kinase kinase β, sirtuin 1
unspecific	Tachiban a	2017	28626056	ischemic	mouse transient and permanent MCAO	*in vivo*	number of vessels and CD34 staining, EM in infarct (Fig. 2)	n/a
unspecific	Takagi	2017	26661252	hemorrhagic	collagenase in human BMVEC	*in vitro*	PI (Fig. 5), rest in BBB model, *in vivo:* no cell death assessed	n/a
unspecific	Yen	2016	26746802	ischemic	transient OGD in primary mouse CEC	*in vitro*	MTT (Fig. 4), *in vivo*: only BBB dysfunction assessed	n/a
unspecific	Yin	2013	23408111	ischemic	permanent OGD in primary mouse cerebral vascular endothelial cells	*in vitro*	MTT, LDH (Fig. 1), in vivo: no stroke or no cell death	KLF11, PPARγ
unspecific	Zhan	2017	28634073	ischemic	transient OGD in rat BMVEC	*in vitro*	MTT (Fig. 2, 4), PI (Fig. 2)	MEG3, NOX4
unspecific	Zhao	2019	31057477	ischemic	transient OGD in primary rat BMECs	*in vitro*	Trypan blue (Fig. 4)	n/a
unspecific	Zhu	2018	30090656	ischemic	transient OGD in bEnd.3	*in vitro*	CCK-8, PI (Fig. 3), *in vivo*: only BBB dysfunction and whole tissue	n/a

Supplemental Table 2. Excluded publications, with the reasons for their exclusion.

No.	First Author	Year	PMID	Other cells used or assessed	Cell death not investigated	Review	Non-English publication	Duplicated data!	Full-text not accessible
1.	Alfieri	2013	24017972		x				
2.	Brait	2019	30582456		x				
3.	Chang	2015	26395442	x					
4.	Chen	2010	20705928		x				
5.	Chen	2017	28965081	x					
6.	Cheng	2019	30775405	x					
7.	Dhanesha	2019	30909835		x				
8.	Du	2019	31064890	x					
9.	Eisa-Beygi	2013	23206891		x				
10.	Fang	2015	26251121		x				
11.	Guo	2015	25627354	x					
12.	Han	2011	21198825	x					
13.	Hansen	2017	28285405	x					
14.	He	2015	25779039	x					
15.	Henry	2013	23103420		x				
16.	Hong	2012	23271289	x					
17.	Hosoo	2017	28655813	x					
18.	Hou	2015	25601765		x				
19.	Hu	2016	27132231		x				
20.	Hu	2013	23262083	x					
21.	Ishikawa	2013	24130140	x					
22.	Ji	2012	22759265		x				
23.	Kuntz	2014	24084699	x					
24.	Lee	2010	19840779	x					
25.	Li	2014	24867613	x					
26.	Li	2013	23801613				x		
27.	Liu	2010	20437588		x				
28.	Liu	2017	28378105		x				
29.	Liu	2019	31169190	x					
30.	Lockman	2012	21935732					x	
31.	Lockman	2012	23099055					x	
32.	Lyu	2018	29773101				x		
33.	Machado-Pereira	2018	29518539	x					
34.	Marbacher	2012	22595025	x					
35.	Mei	2017	28469657	x					

No.	First Author	Year	PMID	Other cells used or assessed	Cell death not investigated	Review	Non-English publication	Duplicated data!	Full-text not accessible
36.	Mishiro	2014	25133692		x				
37.	Otero-Ortega	2019	31141256	x					
38.	Pfeilschifter	2010	20514517	x					
39.	Rubattu	2017	28640254	x					
40.	Ryou	2013	23891792	x					
41.	Shi	2012	23073197				x		
42.	Shi	2017	28137866		x				
43.	Silachev	2016	26742738	x					
44.	Simard	2010	20035575			x			
45.	Sun	2010	20515821	x					
46.	Sun	2014	24503888	x					
47.	Taguchi	2011	20859292		x				
48.	Teng	2018	29802529	x					
49.	Tian	2016	27565895	x					
50.	Toyama	2014	24371084		x				
51.	Vadivelu	2017	28636928		x				
52.	Wang	2018	29575939					x	
53.	Wang	2018	29115440						x
54.	Xi	2017	29042193	x					
55.	Xin	2017	28413461					x	
56.	Xu	2013	24386821				x		
57.	Yen	2013	23930775		x				
58.	Yong	2019	31204565						x
59.	Yougbare	2015	25774504	x					
60.	Yunchang	2015	26923578	x					
61.	Zhang	2013	23690990		x				
62.	Zhao	2018	30488141		x				
63.	Zimering	2010	20570807	x					

Supplemental Table 3. Risk of bias assessment of the included studies. The assessment was performed with respect to the endothelial cell death measurements. Green indicates low risk, red indicates high risk, and yellow indicates unclear risk due to lack of information or uncertainty over the potential for bias. Four studies were biased for incomplete outcome analysis due to unexplained unequal numbers of biological replicates. A common statistical issue identified is the use of statistical tests that require normally distributed data (e.g., *t*-test or ANOVA) without reporting that normal distribution or the homogeneity of variance was tested or confirmed (yellow label). Incorrect statistical tests (red label) were reported when parametric tests were performed with a sample size (n=3-4 per group) insufficient to assume normal distribution or when *t*-tests were used for multiple comparisons. NA: not applicable.

			Selection bias			Performance/ detection bias		Attrition bias	Reporting bias	Other bias	
First Author	Year	PMID	Randomization	Sample size calculation	Allocation concealment	Blinding of researchers	Exposure classification	Complete outcome data	Selective reporting	Conflict of interest	Statistical analysis
Abdul	2018	29909454									
Abdullah	2015	26546149									
Ahmad	2019	31138990									
Bake	2019	30287160			NA						
Basuroy	2013	23576575					NA				
Butt	2011	21356382									
Cao	2016	26915982			NA		NA				
Cardoso	2012	22586454									
Ceruti	2011	21672581					NA				
Chen	2016	27652091			NA						
Chen	2017	28072729									
Chen	2017	28438530									
Chen	2018	29327153									
Chen	2018	29963617									
Cheng	2019	30414726					NA				
Chu	2017	28402976					NA				NA
Clark	2012	23082218			NA		NA				
Cui	2016	26111628									
Cui	2019	30876978									
Dong	2018	30120860			NA						
ElAli	2012	21767321									
Engelhardt	2015	25879623									
Fang	2016	26887441			NA						
Feng	2019	30828041									
Friedrich	2012	22306092									
Friedrich	2013	24250830									
Fu	2014	24930357			NA		NA				
Fu	2019	30962864									
Fumoto	2019	30628008									
Gao	2010	20496198					NA				NA

			Selection bias			Performance/ detection bias		Attrition bias	Report-ing bias	Other bias	
First Author	Year	PMID	Randomization	Sample size calculation	Allocation concealment	Blinding of researchers	Exposure classification	Complete outcome data	Selective reporting	Conflict of interest	Statistical analysis
Garbuzova-Davis	2014	24610730									
Ge	2018	29695199									
Guo	2010	19861973			NA		NA				
Guo	2010	20664263			NA		NA				
Han	2011	21392095									NA
Han	2016	26231971					NA				
Hasegawa	2012	22183833									NA
Hawkins	2015	25669912									
He	2012	22944263									NA
Hou	2011	21722091									
Hu	2015	25543188			NA		NA				
Huang	2016	27592408			NA		NA				
Huang	2019	30518742			NA						
Hunter	2019	31118073									
Hwang	2019	31024439					NA				
Imai	2017	27855593									
Imai	2019	30996325									
Iwata	2018	30092231					NA				
Ji	2013	24028618			NA		NA				
Ji	2017	28603491					NA				
Jiang	2017	27514013									
Jiang	2017	29146880									
Jung	2010	20560878			NA		NA				
Kapitulnik	2012	22811666									
Kokubu	2017	28336435									
Krupinski	2012	23227823					NA				
Ku	2016	27128638									
Ku	2016	27303049									
Kuntz	2014	24333620					NA				NA
Lam	2010	20505729									
Lecht	2010	20012228			NA		NA				
Lee	2018	29870781			NA		NA				
Li	2012	22056225									NA
Li	2012	22472112					NA				NA
Li	2013	23558089									
Li	2014	25070048									
Li	2014	25618978			NA		NA				
Li	2016	26578299									

			Selection bias			Performance/ detection bias		Attrition bias	Report-ing bias	Other bias	
First Author	Year	PMID	Randomization	Sample size calculation	Allocation concealment	Blinding of researchers	Exposure classification	Complete outcome data	Selective reporting	Conflict of interest	Statistical analysis
Li	2017	28433650									
Li	2019	30839193									
Li	2019	30995438									
Li	2019	31290452			NA		NA				
Li	2019	31293373									
Liao	2016	27885275					NA				
Liu	2012	23071504			NA		NA				
Liu	2013	23967200									
Liu	2016	27324700									
Liu	2017	28367097									
Lok	2011	21534958			NA		NA				
Lonati	2019	31370282									
Long	2018	30233065					NA				
Luo	2017	28252551									
Luo	2019	30911000									
Lv	2016	26306919									
Lyden	2018	30461327			NA		NA				
Mao	2018	29533123					NA				
Mey	2013	23816753									
Orsini	2018	30354247					NA				
Page	2016	27724968					NA				
Palmela	2011	21463246									
Palmela	2012	22590454									
Palmela	2015	25821432									
Pang	2016	27463015									
Pang	2017	27796945									
Pang	2018	30622670									
Panickar	2015	24773045					NA				
Park	2013	23374901			NA		NA				
Qi	2016	27388935									NA
Qi	2019	31152817									
Rakkar	2016	26527181									
Ran	2011	21960347									
Redzic	2015	24206924			NA		NA				
Ren	2018	30038058			NA		NA				
Rodriguez	2017	27768124									
Ruan	2019	30607811									
Salvador	2015	26347611			NA		NA				
Shi	2016	26865248									
Shi	2018	30317635									

			Selection bias			Performance/ detection bias		Attrition bias	Report-ing bias	Other bias	
First Author	Year	PMID	Randomization	Sample size calculation	Allocation concealment	Blinding of researchers	Exposure classification	Complete outcome data	Selective reporting	Conflict of interest	Statistical analysis
Shin	2018	29574357			NA		NA				
Song	2010	20125184					NA				
Song	2014	25126203									
Sukumari-Ramesh	2010	20737478									
Sun	2019	30422362			NA		NA				
Sun	2019	30688264									
Tachibana	2017	28626056									
Takagi	2017	26661252					NA				
Tian	2013	23344049									
Tian	2013	23948104									
Tian	2018	28987818									
Tu	2016	26111627									
Wang	2018	29424909									
Wang	2018	30555402									
Wang	2019	30496821									
Wu	2014	24352801									
Wu	2019	30260010									
Xiang	2017	28844675									
Xu	2017	29039513									
Xu	2019	30859754									
Yan	2011	21586287									NA
Yang	2012	25722681									
Yang	2017	27380043									
Yang	2018	29203245									
Yang	2018	30414401									
Yen	2016	26746802			NA		NA				
Yin	2010	20445066									
Yin	2013	23408111			NA		NA				
Yu	2017	29311781									
Yu	2019	31105826									
Zhan	2017	28634073					NA				
Zhang	2016	27630541									
Zhang	2017	28093478									
Zhang	2017	28347817									
Zhang	2018	29377234									
Zhang	2018	29524637									
Zhang	2018	30158991									
Zhang	2018	30419554									
Zhang	2019	30787267									

			Selection bias			Performance/ detection bias		Attrition bias	Report-ing bias	Other bias	
First Author	Year	PMID	Randomization	Sample size calculation	Allocation concealment	Blinding of researchers	Exposure classification	Complete outcome data	Selective reporting	Conflict of interest	Statistical analysis
Zhao	2019	31057477			NA		NA				
Zhou	2014	24482345									
Zhou	2019	30408478									
Zhu	2018	30090656					NA				
Zuo	2018	29439730									

PRISMA Checklist

Section/topic	#	Checklist item	Reported on page #
**TITLE**			
Title	1	Identify the report as a systematic review, meta-analysis, or both.	1
**ABSTRACT**			
Structured summary	2	Provide a structured summary including, as applicable: background; objectives; data sources; study eligibility criteria, participants, and interventions; study appraisal and synthesis methods; results; limitations; conclusions and implications of key findings; systematic review registration number.	2
**INTRODUCTION**			
Rationale	3	Describe the rationale for the review in the context of what is already known.	11
Objectives	4	Provide an explicit statement of questions being addressed with reference to participants, interventions, comparisons, outcomes, and study design (PICOS).	11
**METHODS**			
Protocol and registration	5	Indicate if a review protocol exists, if and where it can be accessed (e.g., Web address), and, if available, provide registration information including registration number.	11
Eligibility criteria	6	Specify study characteristics (e.g., PICOS, length of follow-up) and report characteristics (e.g., years considered, language, publication status) used as criteria for eligibility, giving rationale.	12
Information sources	7	Describe all information sources (e.g., databases with dates of coverage, contact with study authors to identify additional studies) in the search and date last searched.	12
Search	8	Present full electronic search strategy for at least one database, including any limits used, such that it could be repeated.	Supplemental data
Study selection	9	State the process for selecting studies (i.e., screening, eligibility, included in systematic review, and, if applicable, included in the meta-analysis).	12-13
Data collection process	10	Describe method of data extraction from reports (e.g., piloted forms, independently, in duplicate) and any processes for obtaining and confirming data from investigators.	12-13
Data items	11	List and define all variables for which data were sought (e.g., PICOS, funding sources) and any assumptions and simplifications made.	12-13
Risk of bias in individual studies	12	Describe methods used for assessing risk of bias of individual studies (including specification of whether this was done at the study or outcome level), and how this information is to be used in any data synthesis.	12-13
Summary measures	13	State the principal summary measures (e.g., risk ratio, difference in means).	n/a
Synthesis of results	14	Describe the methods of handling data and combining results of studies, if done, including measures of consistency (e.g., I^2) for each meta-analysis.	n/a

Section/topic	#	Checklist item	Reported on page #
Risk of bias across studies	15	Specify any assessment of risk of bias that may affect the cumulative evidence (e.g., publication bias, selective reporting within studies).	25-26
Additional analyses	16	Describe methods of additional analyses (e.g., sensitivity or subgroup analyses, meta-regression), if done, indicating which were pre-specified.	n/a
**RESULTS**			
Study selection	17	Give numbers of studies screened, assessed for eligibility, and included in the review, with reasons for exclusions at each stage, ideally with a flow diagram.	12-13
Study characteristics	18	For each study, present characteristics for which data were extracted (e.g., study size, PICOS, follow-up period) and provide the citations.	Supplemental Table 1
Risk of bias within studies	19	Present data on risk of bias of each study and, if available, any outcome level assessment (see item 12).	Supplemental Table 3
Results of individual studies	20	For all outcomes considered (benefits or harms), present, for each study: (a) simple summary data for each intervention group (b) effect estimates and confidence intervals, ideally with a forest plot.	n/a
Synthesis of results	21	Present results of each meta-analysis done, including confidence intervals and measures of consistency.	n/a
Risk of bias across studies	22	Present results of any assessment of risk of bias across studies (see Item 15).	25-26
Additional analysis	23	Give results of additional analyses, if done (e.g., sensitivity or subgroup analyses, meta-regression [see Item 16]).	n/a
**DISCUSSION**			
Summary of evidence	24	Summarize the main findings including the strength of evidence for each main outcome; consider their relevance to key groups (e.g., healthcare providers, users, and policy makers).	13-22
Limitations	25	Discuss limitations at study and outcome level (e.g., risk of bias), and at review-level (e.g., incomplete retrieval of identified research, reporting bias).	25-26
Conclusions	26	Provide a general interpretation of the results in the context of other evidence, and implications for future research.	26
**FUNDING**			
Funding	27	Describe sources of funding for the systematic review and other support (e.g., supply of data); role of funders for the systematic review.	27

From: Moher D, Liberati A, Tetzlaff J, Altman DG, The PRISMA Group (2009). Preferred Reporting Items for Systematic Reviews and Meta-Analyses: The PRISMA Statement. PLoS Med 6(6): e1000097. doi:10.1371/journal.pmed1000097 For more information, visit: www.prisma-statement.org.
